# An extended period of elevated influenza mortality risk follows the main waves of influenza pandemics

**DOI:** 10.1016/j.socscimed.2023.115975

**Published:** 2023-05-19

**Authors:** Max Schroeder, Spyridon Lazarakis, Rebecca Mancy, Konstantinos Angelopoulos

**Affiliations:** a University of Glasgow, UK; b Lancaster University, UK; c CESifo, Munich, Germany

**Keywords:** Influenza pandemics, Post-pandemic period, Mortality risk dynamics

## Abstract

Understanding the extent and evolution of pandemic-induced mortality risk is critical given its wide-ranging impacts on population health and socioeconomic outcomes. We examine empirically the persistence and scale of influenza mortality risk following the main waves of influenza pandemics, a quantitative analysis of which is required to understand the true scale of pandemic-induced risk. We provide evidence from municipal public health records that multiple recurrent outbreaks followed the main waves of the 1918-19 pandemic in eight large cities in the UK, a pattern we confirm using data for the same period in the US and data for multiple influenza pandemics during the period 1838–2000 in England and Wales. To estimate the persistence and scale of latent post-pandemic influenza mortality risk, we model the stochastic process of mortality rates as a sequence of bounded Pareto distributions whose tail indexes evolves over time. Consistently across pandemics and locations, we find that influenza mortality risk remains elevated for around two decades after the main pandemic waves before more rapid convergence to background influenza mortality, amplifying the impact of pandemics. Despite the commonality in duration, there is heterogeneity in the persistence and scale of risk across the cities, suggesting effects of both immunity and socioeconomic conditions.

## 1 Introduction and background

Historically, influenza pandemics have given rise to periods of drastically increased mortality. These sharp increases have had important implications for individuals via increased mortality and health risk, as well as for wider society through restrictions, pressure on healthcare systems, and economic effects (Cohn, 2007; Dewitte and Wissler, 2022; Maffly-Kipp et al., 2021; Neelsen and Stratmann, 2012; Ogasawara, 2017; Patterson and Pyle, 1991; Reid, 2005; Shaw-Taylor, 2020; van Doren and Brown, 2023; Zuijderduijn and De Moor, 2013). The health and socioeconomic effects of pandemics have been shown to act unequally across the population, leading to concerns that pandemics might amplify pre-existing inequalities, having the worst effects on the most vulnerable groups in society (Clay et al., 2019; Connor et al., 2020; Dewitte and Wissler, 2022; Grantz et al., 2016; Mamelund, 2006, 2018; Marmot and Allen, 2020).

Research on the implications of pandemics has primarily focused on effects during the main pandemic waves (Clay et al., 2019; Connor et al., 2020; Grantz et al., 2016; Maffly-Kipp et al., 2021; Mamelund, 2018; Patterson and Pyle, 1991; Reid, 2005). The medium-run implications for health have also been studied by considering, for example, survivorship in the periods before and after the 1918-19 pandemic (van Doren and Kelmelis, 2022), possible impacts of main wave mortality on sex differentials in mortality (Noymer and Garenne, 2000) and subsequent tuberculosis incidence (Noymer, 2009, 2011; Noymer and Garenne, 2000; van Doren and Sattenspiel, 2021; Zürcher et al., 2016). Studies have also demonstrated that pandemics can have a long legacy (Cohn, 2007; Dewitte and Wissler, 2022; Neelsen and Stratmann, 2012; Noymer, 2009, 2011; Noymer and Garenne, 2000; Ogasawara, 2017; Shaw-Taylor, 2020; van Doren and Sattenspiel, 2021; Zuijderduijn and De Moor, 2013). In this research, the post-pandemic implications arise from the dramatic increase in morbidity and mortality during the main waves, rather than from continued pathogen circulation.

Theoretical reasoning suggests that recurrent outbreaks can follow major epidemics, implying that to understand the full impact of pandemics, outbreaks during the post-pandemic period need to be taken into consideration. Recurrent outbreaks can result from processes such as population turnover, waning immunity, re-introductions of the pathogen, or the emergence of new variants (Anderson & May 1992; Oxford, 2000). Indeed, recent theoretical and modelling research suggests the possibility of recurrent outbreaks following the main waves of COVID-19 (Giannitsarou et al., 2021; Kissler et al., 2020; Lavine et al., 2021; McKie, 2021; Phillips, 2021). Empirically, microbiological research has confirmed that viral descendants of the 1918-19 H1N1 influenza virus did cause influenza outbreaks following the main pandemic waves and were still circulating a century later (Brüssow, 2022; Morens et al., 2009). However, quantitative analysis of the scale of post-pandemic influenza mortality risk associated with recurrent outbreaks of influenza, and the period over which it remains elevated, has attracted little attention in the literature.

We examine the dynamics of influenza mortality rates following historical influenza pandemics. We first analyse the period following the most significant pandemic of the past two centuries, that of 1918-19. We compile a new dataset comprising annual mortality rates from influenza and other respiratory tract diseases using municipal public health records from Medical Officer of Health (MOH) reports for eight major UK cities between 1895 and 1956. We complement this dataset with data for the US during the same period from the Vital Statistics Rates reports of the National Center for Health Statistics (Grove and Hetzel, 1968; Linder and Grove, 1943). We then analyse patterns across pandemics, compiling a continuous time series of annual influenza mortality rates between 1838 and 2000 for England and Wales by combining data from the 20th Century Mortality Files (Office for National Statistics, 2013) for 1901–2000, and from Langford (2002) for the earlier period, both originally based on Registrar General reports. To analyse the persistence and scale of post-pandemic influenza mortality risk, we model the stochastic process of mortality rates following the main pandemic waves as a sequence of bounded Pareto distributions such that the probability of large outbreaks decays over time to background influenza mortality. We then discuss the scale and persistence of recurrent outbreaks in relation to epidemiological and socioeconomic factors.

## 2 Methods

### 2.1 Data on historical post-pandemic mortality rates

We compiled three datasets as summarised below, with further details provided in Section A.1 of Supplementary Information (SI).

First, we constructed a new dataset of mortality rates from influenza and other respiratory tract diseases for eight large municipalities across the UK between 1895 and 1956. We transcribed city-level data from Medical Officer of Health (MOH) annual reports, administrative documents covering a range of public health issues at the municipal level. The first reports begin in the mid-19th century, with more consistent coverage after about 1895, extending to most municipalities in the UK until the early 1970s. We used the annual reports for Belfast, Birmingham, Cardiff, Glasgow, London (London County Council MOH reports), Liverpool, Manchester and Sheffield to compile mortality rates for available years between 1895 and 1956. The reports can be viewed on the Wellcome Collection website, with data taken from tables available in the form of images of the relevant pages (Wellcome Trust, 2021). Annual statistics regarding mortality by cause of death are typically presented in tables within the reports or in their appendices. Where available, we recorded numbers of deaths and population size, and used this to compute cause-specific mortality rates per million (in some cases, only rates were reported, and we recorded these directly). We also transcribed infant mortality rates per 1000 births for each city from 1895 to 1950.

Second, we transcribed influenza mortality rates for the US from annual tables covering the period 1900–1956 contained within the Vital Statistics Rates reports of the National Center for Health Statistics (Grove and Hetzel, 1968; Linder and Grove, 1943).

Third, we compiled influenza mortality rates per million population for England and Wales from two sources. Mortality rates between 1838 and 1910 were taken from Langford (2002) and from 1901 onwards calculated from numbers of influenza deaths and population from the 20th Century Mortality Files (Office for National Statistics, 2013). In both cases, deaths from influenza were originally taken from Registrar General reports; we also checked agreement between the two series for the overlapping period 1901–1910. Infant mortality rates for 1895–1917 were taken from Vital Statistics tables available from the Office for National Statistics (2022a), originally from Registrar General reports.

### 2.2 A model of post-pandemic mortality dynamics

The mortality rates that we observe in the data are the outcome of an underlying stochastic process that governs the probability with which any given mortality rate can arise; that is, there is a distribution of potential mortality rates in any given year, of which one realisation is observed, and this distribution changes over time. Elevated mortality risk in the post-pandemic period is a latent property of this stochastic process and reflects an increased probability of higher mortality rates. To study the time evolution of mortality risk, we need to study changes in relevant properties of the distribution of mortality rates over time.

We model the dynamic evolution of annual influenza mortality rates after the main pandemic waves as outcomes drawn from a sequence of bounded Pareto distributions. We choose this distribution because the size of outbreaks in a given year has been shown to be highly over-dispersed and well modelled by a fat-tailed distribution such as the bounded Pareto (Cirillo and Taleb, 2020). We assume that the inverse of the tail index of the bounded Pareto distributions after the main waves of the pandemic evolves following an exponential function, allowing mortality risk to decline towards background mortality during the period until the emergence of the next pandemic, which forms a natural bound to each post-pandemic period.

During the period 1838–2000, there were recognised pandemics in 1847-48, 1890-91, 1918-19, 1957-59 and 1968-70 (Hill et al., 2017; Pandemic Influenza Preparedness Team and UK Department of Health, 2011) (see SI Section A.2 for more discussion regarding the choice of pandemics). Given this, we denote the influenza mortality rate in year *t* by *d_t_*, for *t* = 0,1, 2, …, *N*, where the time period refers to (1920, 1921,…, 1956) for the eight cities in the UK, England and Wales, and the US with reference the 1918-19 pandemic, and for England and Wales, also to (1849, 1850,…, 1889) with reference to the 1847-48 pandemic, to (1892, 1893,…, 1917) with reference to the 1890-91 pandemic and to (1971, 1972,…, 2000) with reference to the 1968-70 pandemic. Because of the short time period between 1957 and the next pandemic in 1968, we do not consider the 1957-59 pandemic in the statistical analysis.

The mathematical form of the model describes influenza mortality rates in each year as being drawn from

(1) $$d_{t}∼PD_{t}\left(d_{l},d_{u},\alpha_{t}\right),$$

where *PD_t_* denotes the bounded Pareto distribution in period *t, α_t_* > 0 is the time-varying Pareto tail index (also referred to as the shape parameter), and *d_l_* > 0 and *d_u_* > *d_l_* are, respectively, lower and upper bounds. Defining *η_t_* = ^1^/*α_t_*, we assume the time process

(2) $$\eta_{t}=e^{\eta_{0}}\;e^{-\lambda t},$$

where *λ* > 0 determines the shape of the exponential decay of the inverse of the tail index over time, while *η*_0_ sets the initial level of the probabilities. Together, these determine the sequence ${\left(\alpha_{t}\right)}_{t=0}^{N}$. In this specification, elevated mortality risk, as measured by the probability of higher mortality rates, is a decreasing function of the Pareto tail index, and thus declines over time as the Pareto tail index increases. Further discussion of the modelling decisions can be found in Section B.1 of SI.

We fit our model to the influenza mortality rates for each geographical unit and pandemic conditional on its corresponding main wave and background mortality. We use the parameters *d_u_* and *d_l_* to capture these following the logic that during the post-pandemic period, influenza mortality cannot be higher than main wave mortality and cannot fall below the lowest mortality observed during the series. We then obtain *λ* and *η*_0_ using maximum likelihood methods, showing that the maximum likelihood estimator performs well for our samples (see Sections B.2-B.4 of SI for more detail).

## 3 Results

### 3.1 Post-pandemic influenza mortality

**Fig. 1:**
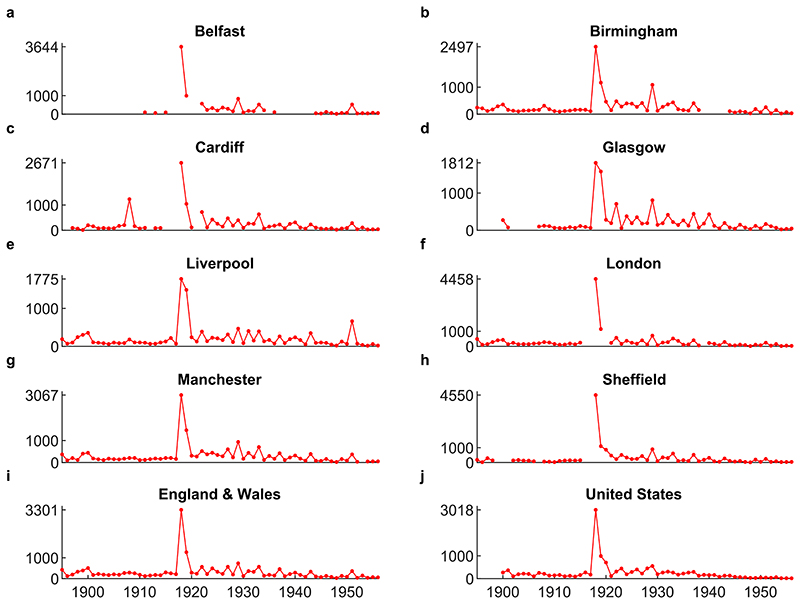
Annual mortality rate from influenza in deaths per million population. The upper y-axis tick indicates the maximum mortality rate for each data series. See Section 2.1 for data sources.

Fig. 1 shows the time series of annual mortality rates from influenza across the geographies we study from 1895 to 1956 (i.e. until the 1957–1959 influenza pandemic). During this period, there was only one influenza pandemic, that of 1918-19, which is also generally accepted as the most important influenza pandemic during the longer period we study. The most striking feature in Fig. 1 is indeed the massive increase in mortality during the main pandemic waves.

The key new insight from the time series in Fig. 1 is that, across the different geographies, we observe frequent influenza outbreaks for an extended period following the 1918-19 pandemic, which eventually died out. Focusing on the eight municipalities (a)-(h), we observe multiple spikes of high mortality over the decades following the main pandemic waves, several of which are higher than the mortality in any year during the pre-pandemic period. Despite geographic variation in the scale of these outbreaks, the pattern is similar across all cities. The same pattern emerges in the data for England and Wales (i) and for the US (j). Recent microbiological research has linked the causal agent of some of these epidemics to viral descendants of the 1918-19 strain (Brüssow, 2022; Morens et al., 2009; see also SI Section B.9 for more discussion of the epidemiology of the 1918-19 pandemic and subsequent outbreaks).

The spikes of high mortality that we observe in the data were acknowledged by contemporary experts as influenza epidemics or large outbreaks. For example, the Medical Officers for London, Glasgow and Liverpool explicitly refer to many of the mortality spikes seen in Fig. 1 as influenza epidemics. Almost all annual reports for these cities contain some discussion of influenza. We searched for the word ‘influenza’ among the available London County Council MOH reports between 1920 and 1957, finding that the reports for 1922, 1927, 1929, 1933, and 1937 identify these years as influenza epidemics. We then cross-checked these years in the reports for Liverpool and Glasgow. The reports for Liverpool also comment on these years as having outbreaks or epidemics of influenza. For Glasgow, 1926 rather than 1927 is noted as an epidemic year, while the ‘1933’ epidemic started in the city in December 1932 and is discussed as an epidemic in that report. The language and discussion in the MOH reports demonstrate that epidemics in these years were understood as major influenza episodes rather than interannual variation. Further, the epidemics that affected the UK in 1922, 1928-29, and the mid-30s have also been noted in other countries (Brüssow, 2022; Taubenberger and Morens, 2006). This is also discussed in the MOH reports: the 1921 London report explains that the 1921-22 epidemic was seen throughout Europe at the end of 1921, while the Liverpool 1922 report notes that the epidemic was “common with a large part of the civilised world” (p32); the 1929 Glasgow MOH report comments that this epidemic came to Glasgow from the United States.

To illustrate the importance of post-pandemic outbreaks for influenza mortality, we show average mortality during the main waves and the surrounding decades in Table 1. The 1898–1907 decade is very close to the earlier 1890-91 pandemic, so we focus our analysis on the last pre-pandemic decade and the two decades that follow the main waves. Relative to the pre-pandemic decade 1908–1917, the mortality during the first post-pandemic decade of the 1918-19 pandemic is around 2–4 times higher. Even in the second decade after the main pandemic waves, mortality is higher in all cities except Cardiff, and nearly double on average. In fact, outbreaks during the ten years following the pandemic resulted in nearly as many deaths as the main waves (this can be seen by comparing total mortality during 1918-19 to total mortality during the 1920-29 period). Taken individually, the size of these outbreaks was considerable, reaching a mortality of around 500–1000 per million for each geography, with the range also demonstrating variation between geographies. It is also useful to contextualise the scale of recurrent outbreaks in the cities relative to the size of the main waves. In this sense, the three cities with relatively low main wave mortality, Liverpool, Glasgow and Birmingham, experienced very large recurrent outbreaks, at around a third of the size of peak main wave mortality. Indeed, relative to their main wave mortality, these recurrent outbreaks were larger or comparable to those in London and Sheffield, which experienced very high main wave mortality. Finally, we note that the series for England and Wales demonstrates generally higher influenza mortality than the cities (even when computing the average of the cities weighted by the population; see Table A1 in SI Section A.3), but the gap closes during the period after the 1918-19 pandemic. That the gap closes may suggest that influenza mortality induced by this pandemic (during the main waves and subsequent recurrent outbreaks) was more important in the large cities, relative to earlier pandemics (see additional discussion in SI Section A.3).

In addition to deaths recorded as influenza, the MOH noted that the influenza epidemics we study had an impact on deaths from other diseases, most notably pneumonia and bronchitis, which often occurred as secondary infections or ‘complications’ of influenza. From the data, it is not possible to decompose deaths recorded as being due to these other respiratory tract infections into those resulting from influenza and those caused by other factors. The non-influenza factors, which are not related to our analysis, are likely to constitute the majority of deaths from pneumonia and bronchitis. These diseases demonstrate a secular downward trend during the period of our analysis (see Section A.1 of SI for more detail), largely as a result of improvements in public health and medicine over this period. In contrast, influenza mortality exhibits only a temporary downward trend following the pandemic, reflecting the return to background mortality (Fig. 1 shows that influenza mortality does not exhibit a noticeable trend prior to the 1918-19 pandemic). To avoid problems of conflated causes and secular trends, we proceed with the statistical analysis of post-pandemic influenza mortality risk based on reported influenza mortality rates. Nonetheless, analysis of mortality from the other respiratory tract diseases (see Section A.4 of SI) shows that for each epidemic during the post-pandemic decades, excess mortality due to influenza was highly correlated, across the cities, with excess mortality due to the broader category of respiratory tract infections. This finding suggests that the variation in influenza mortality across cities associated with recurrent outbreaks, which we analyse later, should not depend critically on our decision to focus on influenza for our analysis.

**Fig. 2:**
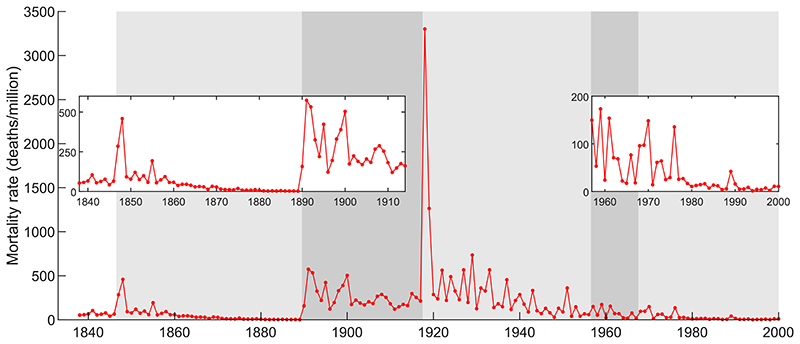
Annual mortality rate from influenza in England and Wales in deaths per million population. Alternating grey shading delimits the periods associated with each pandemic (e.g. the first light grey area corresponds to the 1847-48 pandemic and subsequent post-pandemic period; see Table 2 for pandemic dates). Because the exceptionally high 1918 mortality sets the scale of the figure, insets show the periods until 1914 and from 1957 scaled to mortality relevant for these periods. See Section 2.1 for data sources.

Expanding the period of analysis allows us to consider additional pandemics. Fig. 2 shows the mortality rate from influenza in England and Wales over the period 1838–2000, and Table 2 shows average mortality during the main waves and the surrounding decades. The main message from Fig. 2 is that the pandemics we consider were all followed by an extended period of frequent outbreaks, some of which were large relative to the main waves. This implies that the general pattern seen in Fig. 1 holds not only across geographies but also across pandemics. The more recent pandemics of 1957-59 and 1968-70 and their recurrent outbreaks resulted in lower mortality than the pandemics of the 19th and early 20th centuries (see SI Section B.9 for more discussion of the epidemiology of influenza). Nonetheless, the post-pandemic period still demonstrates multiple outbreaks, and the size of these relative to the main waves is similar to or even greater than those of earlier pandemics. This can be seen by computing the ratio of post-pandemic mortality to main wave mortality, both for averages and maximum values.

### 3.2 The dynamics of post-pandemic influenza outbreak risk

The recurrent outbreaks observed in the data during the post-pandemic period imply a period of elevated influenza mortality risk. To quantify relevant properties of this risk, we fit the model described in Section 2.2 separately to all geographies and pandemics we study (see Section B.2 in SI for more detail and for parameter estimates).

To present our results, we focus on the time evolution of the probability of elevated mortality rates. In particular, we calculate the probability that the mortality rate exceeds a specified threshold in a given year, a quantity that we refer to as outbreak risk. To achieve this, we use the estimated model parameters to construct the time series of distributions of mortality rates in the decades following the main pandemic waves and calculate the probability of elevated mortality rates from these. We then plot the time evolution of the probability that the mortality rate exceeds thresholds of 500 deaths per million and 750 deaths per million. The 500 per million threshold is comparable to peak mortality during the preceding pandemics of 1847-48 and 1890-91, and over three times that of peak mortality of the pandemics of 1957-59 and 1968-70. For the period after 1920, this threshold also identifies as outbreaks those years described as having exceptionally high influenza mortality in the MOH reports. The 750 per million threshold corresponds to around a third of the mortality during the main waves of the 1918-19 pandemic. More generally, these thresholds identify large disease outbreaks that were *possible* given the dynamic process for mortality risk, even if unrealised *ex post*. The two thresholds also roughly correspond to COVID-19 mortality in the UK during, respectively, the Delta wave (April–November 2021; ~450 deaths per million) and the Omicron wave (December 2021–February 2022; ~867 deaths per million) (Office for National Statistics, 2022b).

**Fig. 3:**
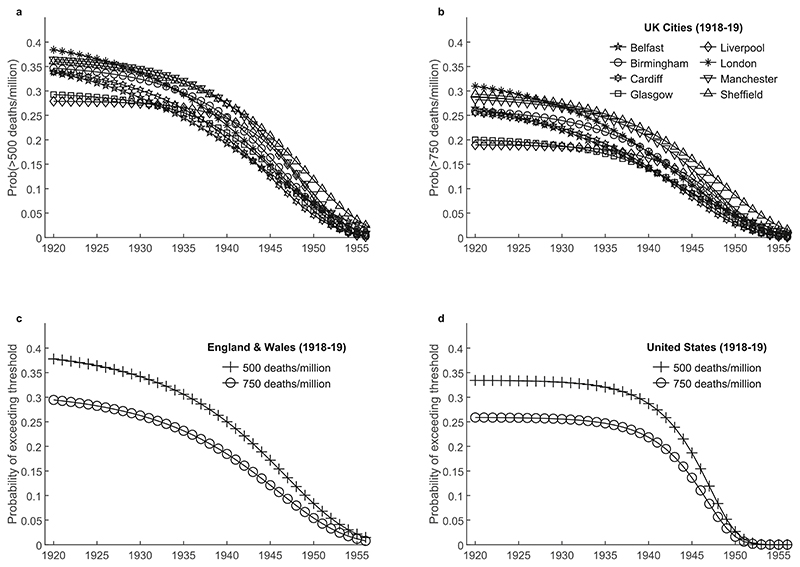
Time evolution of outbreak risk following the 1918-19 pandemic. (a)–(b) Outbreak risk computed from the model fitted to each city, and for thresholds of (a) 500 deaths per million population and (b) 750 deaths per million population. (c)–(d) Outbreak risk computed from the model fitted to data for England and Wales and the United States, for two thresholds.

Fig. 3 shows the probability of mortality rates exceeding 500 deaths per million and 750 deaths per million over the period 1920-56. For both thresholds, following the 1918-19 pandemic, outbreak risk starts relatively high and declines slowly during the first two decades before more rapid convergence to background mortality. Despite variation across geographies in the rate of decline of risk during these two decades, to which we return later, we note that the duration of the period of especially elevated mortality risk and relatively slow decline is around two decades. During this period, the probability of outbreaks exceeding 500 deaths per million is roughly in the range of 20%–40% and even the threshold of 750 deaths per million has a probability of being exceeded in the range of 15%–30%.

**Fig. 4:**
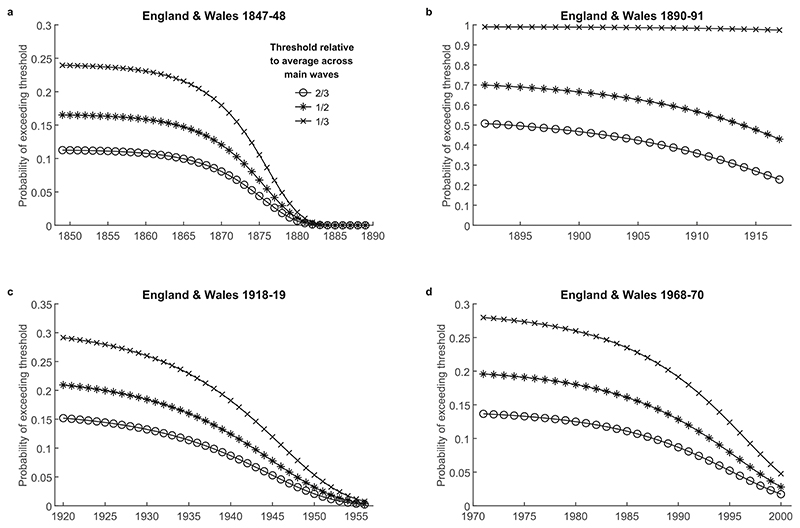
Time evolution of outbreak risk following the 1847-48 and 1890-91, 1918-19 and 1968-70 pandemics in England and Wales, for different thresholds corresponding to proportions of the maximum mortality observed during the main waves of the corresponding pandemic.

Focusing on England and Wales, Fig. 4 shows the time evolution of the probability of outbreaks exceeding different thresholds following the four pandemics we analyse. To make the results comparable across pandemics which differ in their mortality implications, we define thresholds using proportions of the maximum mortality observed during the main waves of the corresponding pandemic. The results indicate that for the same geographic unit, the general pattern of elevated and persistent post-pandemic mortality risk is broadly similar across pandemics spanning two centuries and demonstrates the same qualitative properties as those seen in Fig. 3. We note exceptionally high persistence of influenza mortality risk after the 1890-91 pandemic, as discussed in more detail in SI Section B.5.

**Fig. 5:**
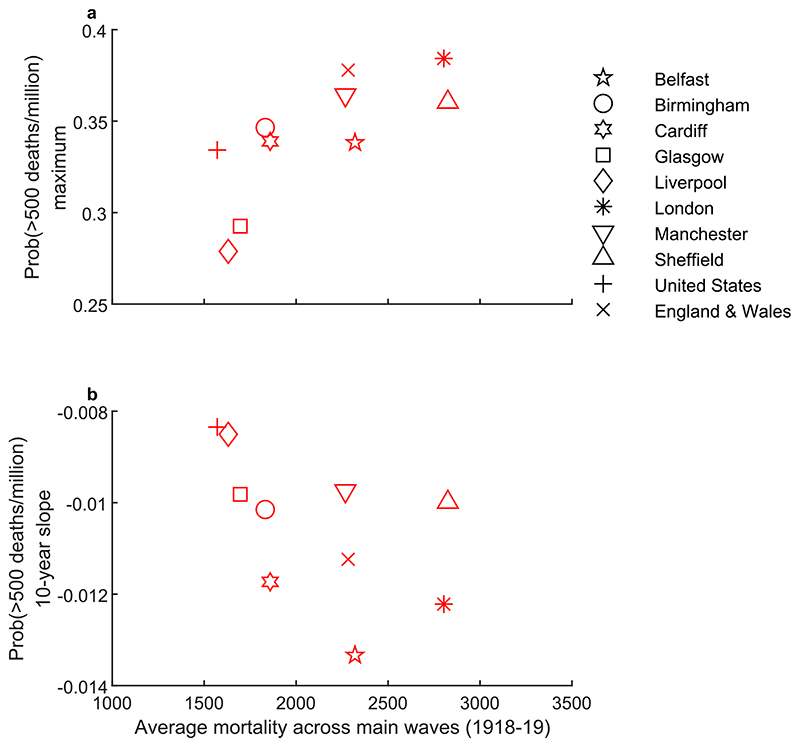
Scatterplots showing (a) the maximum outbreak risk plotted against main wave mortality and (b) the average slope of the outbreak risk over the first post-pandemic decade. In both panels, main wave mortality is measured as the average mortality per million population over the main waves of the 1918-19 pandemic, and the outbreak risk mortality threshold is 500 per million.

Despite the common duration of elevated influenza outbreak risk seen in Fig. 3, there is nonetheless variation in the scale and persistence of risk across geographies following the 1918-19 pandemic. Focusing on the 500 per million threshold and the first post-pandemic decade, we capture the scale of risk as the maximum probability of exceeding this threshold and the persistence over the first decade by computing the average slope over this decade of the curves in Fig. 3, noting that a more negative slope implies a faster decline. Fig. 5 shows these quantities plotted against mortality averaged over the main pandemic waves (in SI Section B.6, we show similar results for the 750 per million threshold and for the second decade, which show broadly similar patterns). The observed relationships suggest that for geographies where main wave mortality was higher, outbreak risk was also higher but less persistent.

## 4 Discussion

### 4.1 Persistence of post-pandemic mortality risk

Our analysis of influenza mortality shows that influenza pandemics have been followed by a long period of recurrent outbreaks. We thus provide empirical evidence to confirm modelling and theoretical work in epidemiology (Giannitsarou et al., 2021; Kissler et al., 2020; Lavine et al., 2021; McKie, 2021; Phillips, 2021). The time series of mortality rates in Section 3.1 reveal that, across locations, geographic scales and pandemics, the main pandemic waves were followed by multiple sizeable influenza outbreaks in the subsequent period, in some cases doubling the death toll of the pandemic over the following decade. Therefore, omitting recurrent outbreaks substantially underestimates the effects of the pandemic. The deadliest recurrent outbreaks followed the deadliest pandemic, that of 1918-19. Indeed, as Tables 1 and 2 reveal, for this pandemic, average annual mortality rates during the first post-pandemic decade approached the main wave mortality of the influenza pandemics of the previous century and were around double the maximum rates seen during the later pandemics of 1957-59 and 1968-70 in England and Wales.

Our statistical model of mortality dynamics allows us to go beyond the patterns observed directly from the data by quantifying the time evolution of the underlying recurrent outbreak risk. This analysis shows that the annual probability of influenza outbreaks exceeding approximately one third of main wave mortality remains high for nearly two decades, before more rapid convergence to background influenza mortality. Moreover, our results show that this pattern and its duration are broadly consistent across the different pandemics and geographies (see SI Section B.5 for further discussion of the idiosyncrasies of mortality after the 1890-91 pandemic).

Our results contribute to research on post-pandemic pathogen recurrence over the long run. For example, existing research has described outbreaks following historical pandemics (Brüssow, 2021, 2022; Brüssow and Brüssow, 2021) and has linked outbreaks following the 1918-19 pandemic to the viral descendants of the original strain, using epidemiological and microbiological methods (Brüssow, 2022; Saglanmak et al., 2011). Our work provides a quantitative analysis of the dynamics of recurrent outbreaks and the risk underpinning them, over the long run. The long time series that we constructed reveals that elevated risk persisted for an extended period, and that full return to background influenza mortality occurred later still. Because outbreaks are inherently stochastic and risk changes slowly, a longer time series is required to observe these patterns. The extended period of recurrent outbreaks raises interesting questions about whether pandemic pathogens should be considered endemic relatively soon after the main waves once they settle into patterns that resemble those of non-pandemic strains (e.g. seasonal patterns, as hinted at by Morens and Fauci, 2007), once they are self-sustaining within a population (Viana et al., 2014) or only much later when incidence is no longer declining (Katzourakis, 2022). Further discussion of implications for epidemiology is in SI Section B.9.

We have focused on analysis of influenza mortality during the post-pandemic period. However, influenza epidemics also have effects on mortality from other related diseases and/or morbidity. Analysis of comparisons in mortality rates from diseases other than influenza between the periods preceding and following historical pandemics is confounded by the general decline in mortality, especially of mortality due to infectious diseases, during the epidemiological transition. For example, there is ongoing discussion about whether pandemics hastened the long run decline of tuberculosis in various locations, primarily for the 1918-19 pandemic (Noymer, 2009, 2011; Noymer and Garenne, 2000; van Doren and Sattenspiel, 2021), with some including the 1890-91 pandemic (Zürcher et al., 2016). There is also research focused on differences between mortality rates from a range of causes during the periods before and after the 1918-19 pandemic (e.g. van Doren and Kelmelis, 2022). We have therefore focused our analysis on influenza mortality, which does not show secular declines over the period of our analysis (see Section 3.1). Indeed, with respect to the 1918-19 pandemic, mortality during the first post-pandemic decade was around 2–4 times higher than during the pre-pandemic decade across the different geographies we study. The increased influenza mortality risk that we document following the influenza pandemics probably also led, other things equal, to increased risk of pneumonia and bronchitis arising as complications of influenza, as is acknowledged in the literature where influenza is often studied in conjunction with one or both of these (e.g. Clay et al., 2019; Grantz et al., 2016; Saglanmak et al., 2011). Like most studies in this literature, we have analysed the implications of pandemics for mortality rather than morbidity. Clearly, elevated mortality risk implies elevated morbidity risk; however, the scale of morbidity risk relative to the main pandemic waves may differ from that of post-pandemic mortality risk, and may even be higher if the pandemic strains become less virulent or treatment and prevention improve over the post-pandemic period (see also SI Section B.9).

Whether the key patterns observed historically can be expected to recur for COVID-19 and future pandemics depends on many factors. For example, even if the underlying epidemiological processes are similar, their impact on population health may differ; levels of medical progress and institutional organisation in the 21st century have allowed governments to implement sophisticated test-and-trace systems and instigate massive lockdowns, and the response to COVID-19 has demonstrated the rapidity with which vaccines can be developed. These vaccines have been, if anything, slightly more effective at preventing severe infections than at interrupting transmission and have typically proven less effective against new variants (Mohammed et al., 2022). Even if mortality rates are reduced, continued circulation of infection can substantially impact public health via increased morbidity and pressure on health systems. Furthermore, the increase in the global population by approximately a factor of four between 1918-19 and 2020 increases the potential for new variants to arise within infected hosts.

Overall, our findings imply that pandemics give rise to a persistent increase in health risk, which is consistent with research that has examined the broader medium run and longer run impacts of pandemics. For example, there is research on *in utero* and early childhood exposure to influenza during 1918-19 on both health (Ogasawara, 2017) and much later socioeconomic outcomes such as educational attainment and marriage (Neelsen and Stratmann, 2012) or both health and socioeconomic outcomes (Almond, 2006). Increased health risk during pandemics has also been linked to deterioration in mental health (e.g. Maffly-Kipp et al., 2021); indeed, for every death, bereavement typically affects several individuals, and mortality impacts on others can therefore be expected to be extensive (Alburez-Gutierrez, 2022). Furthermore, excess mortality puts substantial pressure on public health systems, as observed both historically and during COVID-19 (e.g. van Doren and Brown, 2023), leading to delays in treatment of other illnesses, or reductions in resources available for prevention and cure.

### 4.2 Variation in risk and socioeconomic conditions

Beyond the general pattern and duration of recurrent outbreaks and implied risk, which is consistent across the different influenza pandemics and geographies, we find differences in the magnitude of these outbreaks and in the scale and persistence of implied recurrent outbreak risk.

**Fig. 6:**
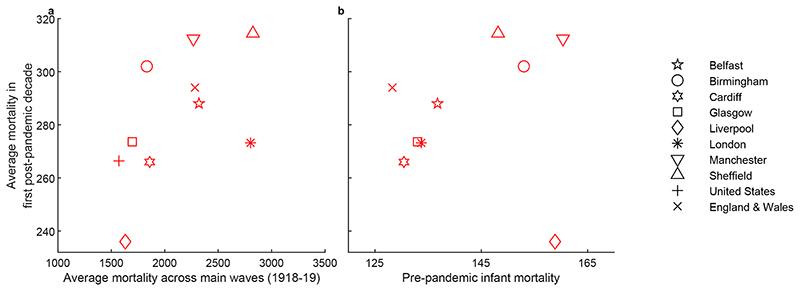
Scatterplots showing the relationship between average influenza mortality during the first post-pandemic decade and (a) the average influenza mortality rate per million population during the main waves and (b) pre-pandemic all-cause infant mortality as deaths per 1000 births (averaged over 1895-1917).

We start with differences observed in the post-pandemic influenza mortality data. Analysis for England and Wales in Section 3.1 reveals that across pandemics, mortality due to recurrent outbreaks is positively associated with the death rates during the main pandemic waves (see Table 2). This relationship probably reflects the importance of strain virulence and any public health and medical advances that apply similarly to the main waves and to the following decade. Across geographies, for the pandemic of 1918-19, the results in Table 1 (also visualised in Fig. 6a), imply that influenza mortality during the first post-pandemic decade is positively associated with the magnitude of the main pandemic waves. This positive relationship suggests that factors that explain variation in mortality between cities during the main waves also apply during the post-pandemic period and scale mortality during it accordingly. These factors are likely to include socioeconomic conditions, and existing research has indeed shown that mortality and morbidity during the 1918-19 main waves were related to socioeconomic conditions (Clay et al., 2019; Grantz et al., 2016; Mamelund, 2006, 2018; Murray et al., 2006; Pearce et al., 2011). Furthermore, a positive relationship between post-pandemic influenza mortality and pre-pandemic infant mortality (from all causes) is also observed in our sample (Fig. 6b). Infant mortality is strongly linked to worse socioeconomic conditions (Pamuk, 1988) and has been used as a proxy in work on heterogeneities in mortality outcomes during the 1918-19 pandemic (Clay et al., 2019) (see also SI Section A.5 for this relationship in Glasgow and London for the period of our analysis).

**Fig. 7:**
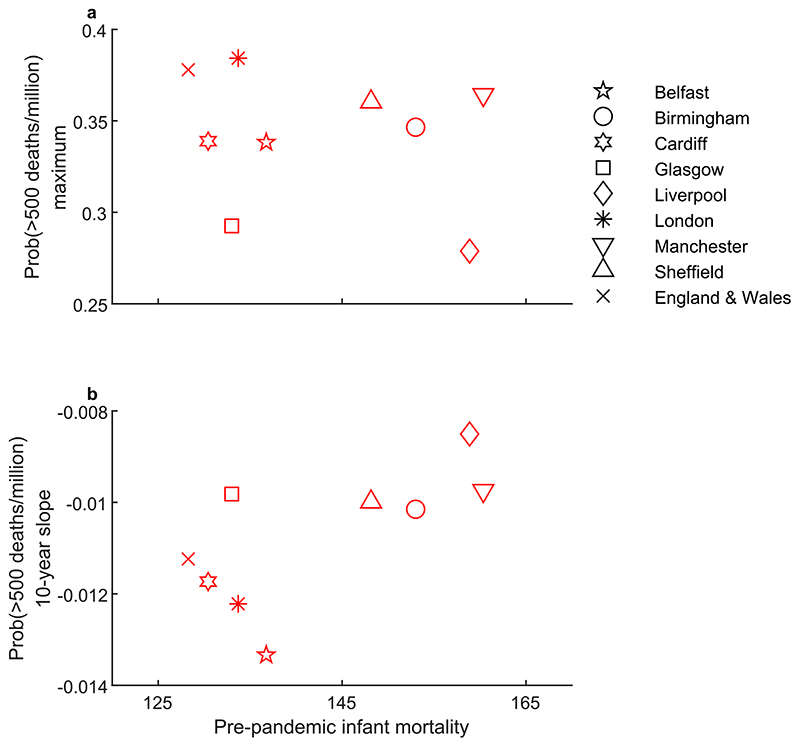
Scatterplots showing (a) the maximum outbreak risk and (b) the average slope of the outbreak risk over the first post-pandemic decade, plotted against pre-pandemic all-cause infant mortality as deaths per 1000 births (averaged over 1895–1917).

Turning to variation in the scale of post-pandemic outbreak risk, Fig. 5a shows a positive relationship between the maximum probability of recurrent outbreaks exceeding 500 deaths per million and main wave mortality. This pattern is similar to that observed in the relationship between post-pandemic influenza mortality during the first decade and main wave mortality (Fig. 6a). In contrast, the maximum probability of recurrent outbreaks exceeding 500 deaths per million shows no obvious relationship with pre-pandemic infant mortality (Fig. 7a). Nonetheless, as discussed below and shown in Fig. 7b, socioeconomic factors do matter for recurrent outbreak risk via persistence. In SI Section B.6, we show that the patterns in Fig. 7 are similar using the 750 per million threshold and the second post-pandemic decade.

The persistence of post-pandemic outbreak risk over the first post-pandemic decade is negatively related to main wave mortality (Fig. 5b) but positively related to infant mortality (Fig. 7b). The first of these relationships is consistent with the explanation that build-up of population-level immunity driven by higher incidence during the main waves should curtail outbreaks and lead to a faster decline in risk. Note that we do not observe a negative relationship between the scale of main wave mortality and post-pandemic mortality in the data (e.g. Fig. 6a). However, our statistical analysis allows us to uncover this immunity effect by distinguishing persistence from the scale of outbreak risk, as approximated by the maximum probability. The positive relationship between persistence and pre-pandemic infant mortality (Fig. 7b) suggests that socioeconomic factors are also important and that it is via persistence of risk that worse socioeconomic conditions contribute to higher post-pandemic mortality. In other words, socioeconomic conditions work to mitigate the beneficial effect of immunity gains.

Factors beyond immunity and socioeconomic conditions may also influence the persistence of recurrent outbreaks. A natural candidate is proximity to the main hub, which for the cities we analyse, is London. Given the development of the railway network in the preceding period (You et al., 2022), higher connectivity to this hub might have driven higher persistence due to more frequent re-introductions. Indeed, epidemiological research underlines the importance of re-introductions for persistence (Mancy et al., 2022). For example, considering the two cities with the highest main wave mortality other than London, we note that Sheffield had more persistent recurrent outbreak risk than Belfast. This finding may be related to differences in socioeconomic conditions associated with infant mortality (see Fig. 7b); however, its relative proximity to London may have contributed. Similarly, considering the two cities with the lowest main wave mortality, Glasgow and Liverpool, Liverpool demonstrates higher persistence, consistent with both the socioeconomic conditions associated with infant mortality (see Fig. 7b) and relative proximity to London.

Overall, our findings suggest that elevated post-pandemic influenza mortality risk is not distributed equally across the population, being a function of socioeconomic conditions. This finding is consistent with a large literature that documents health inequalities during the pandemic and the post-pandemic period, commonly arising as a result of pre-existing income and socioeconomic inequalities (see Dewitte and Wissler, 2022, for a review). For example, several research studies have shown that main wave mortality during the 1918-19 pandemic was related to pre-existing socioeconomic conditions (e.g. Clay et al., 2019; Grantz et al., 2016; Mamelund, 2006, 2018; Murray et al., 2006; Pearce et al., 2011). The health inequality implications of the COVID-19 pandemic and its post-pandemic period have been extensively analysed (Angelopoulos et al., 2021; Connor et al., 2020; Fiske et al., 2022; Mamelund and Dimka, 2021; Marmot and Allen, 2020). Our findings contribute to this literature by suggesting another channel via which pre-existing socioeconomic conditions contribute to health inequalities, namely via an unequal distribution of post-pandemic recurrent outbreak risk.

A prolonged increase in mortality risk and, more broadly, in the health risk associated with it, especially when it is unequally distributed across the population, can also contribute to income inequality. Health shocks, and worse health at the individual level more generally, negatively affect labour market participation and income (Capatina, 2015; Gertler and Gruber, 2002; Jones et al., 2020; Wagstaff, 2007). At the household level, the death of a working parent is probably the biggest labour income shock, but this research shows that major illness can also have negative implications. Negative health shocks to parents can also affect future labour income potential for the offspring via a negative impact on education, resulting from a reduction in household income and perceived trade-offs regarding education (Alam, 2015; Bratti and Mendola, 2014). Therefore, an increase in health risk increases labour income risk, and thus variation in income from labour across the population, which also tends to increase wealth inequality given the positive relationship between labour income risk and wealth inequality (Aiyagari, 1994; Angelopoulos et al., 2020). Health risk and negative health shocks can also affect savings directly. On the one hand, health shocks imply a resource outlay in the form of required medical expenditure (Gertler and Gruber, 2002; Wagstaff, 2007), reducing potential for savings (Angelopoulos et al., 2021; Capatina, 2015). On the other hand, increased health risk increases incentives for savings, as a precaution against future income drops and medical expenses (Angelopoulos et al., 2021; Capatina, 2015; De Nardi et al., 2009). Therefore, an increase in health risk given an unequal distribution of income and wealth tends to increase wealth accumulation for those with higher income and wealth (prior to the increase in health risk) via precautionary savings incentives, but it tends to decrease income and savings for those with lower income and wealth prior to the increase in health risk; overall, it would generate increased income inequality even if the increase in health risk was symmetric across the population. However, because the increase in health risk – and its negative income implications via reduced labour income and increased medical expenditure – are not symmetric, affecting predominantly those with lower income and wealth, the negative effects on income inequality can be even greater.

## 5 Conclusions

Our main result is that pandemic-induced changes in influenza mortality risk are long lasting. In particular, we find that the annual probability of influenza outbreaks exceeding approximately one third of main wave mortality remains high for nearly two decades before more rapid convergence to background influenza mortality. This finding is confirmed for four historical pandemics in England and Wales, and for the 1918-19 pandemic, across geographic units spanning different spatial scales and parts of the world. This extended period of post-pandemic influenza mortality risk amplifies the impact of pandemics and needs to be taken into account in assessing the true scale of the risk they generate. A prolonged increase in mortality risk impacts health and wellbeing directly and indirectly, typically in ways that are unequal. Pandemic-induced increase in health risk, which should include the elevated risk during the period that follows the main pandemic waves, increases health inequality both because it reflects pre-existing socio-economic conditions and because it triggers mechanisms that increase income inequality which ultimately feeds back to further increase in health inequality and its persistence (Angelopoulos et al., 2021).

Historical experience shows that there is the potential for an extended period of recurrent outbreaks following pandemics, and the increased population density and global connectivity of the modern world may amplify this effect. Our results underline the importance of pandemic mitigation planning, including ongoing prevention and pre-paredness during the post-pandemic period.

## Supplementary Material

Supplementary information for this article can be found online at https://doi.org/10.1016/j.socscimed.2023.115975.

Supplementary Information (Appendix A)

## Figures and Tables

**Table 1:** Average annual mortality rate per million population during the 1918-19 pandemic and the surrounding decades for different geographies. Ranges of annual values shown in parentheses.

	1898–1907	1908–1917	1918–1919	1920–1929	1930–1939	1940–1949
Belfast	–	85 (63–98)	**2320** **(996–3644)**	370 (157–831)	210 (92–520)	59 (22–121)
Birmingham	179 (102–358)	154 (94–312)	**1832** **(1167–2497)**	405 (133–1087)	247 (125–436)	96 (30–189)
Cardiff	114 (13–205)	290 (72–1231)	**1859** **(1047–2671)**	315 (113–722)	222 (72–635)	112 (25–314)
Glasgow	150 (77–273)	83 (54–120)	**1696** **(1580–1812)**	334 (60–806)	221 (76–443)	129 (33–431)
Liverpool	152 (61–356)	114 (68–220)	**1631** **(1487–1775)**	240 (114–467)	195 (76–403)	129 (23–349)
London	233 (140–431)	187 (110–281)	**2803** **(1147–4458)**	333 (130–710)	245 (67–520)	125 (23–290)
Manchester	204 (112–440)	170 (112–206)	**2268** **(1468–3067)**	431 (234–934)	290 (115–702)	150 (23–385)
Sheffield	131 (81–169)	102 (24–153)	**2825** **(1100–4550)**	436 (165–890)	249 (85–604)	123 (12–312)
United States	213 (102–365)	156 (89–270)	**1571** **(705–3018)**	316 (113–551)	223 (127–306)	80 (31–158)
England & Wales	263 (169–504)	209 (120–297)	**2283** **(1265–3301)**	385 (196–734)	264 (118–567)	143 (29–333)

**Table 2:** Average annual mortality rate per million population during the main waves of pandemics and the surrounding decades for England and Wales. Ranges of annual values are shown in parentheses.

	Preceding decade	Main waves	Post-pandemic decade I	Post-pandemic decade II
1847–48	65 (42–104)	372 (285–459)	94 (55–193)	39 (14–58)
1890–91	4 (2–7)	366 (157–574)	321 (122–533)	208 (120–288)
1918–19	209 (120–297)	2283 (1265–3301)	385 (196–734)	264 (118–567)
1957–59	104 (29–361)	125 (53–173)	64 (17–154)	55 (14–148)
1968–70	68 (17–173)	114 (96–148)	41 (10–135)	14 (4–42)

## Data Availability

Code and data are available at https://zenodo.org/record/7995423.

## Abbreviations

MOH: Medical Officer of Health
