# Supplementary material for: An extended period of elevated influenza mortality risk follows the main waves of influenza pandemics
Source: Soc Sci Med. Author manuscript; Available in PMC 2023 Aug 9. (PMC7614920; doi:10.1016/j.socscimed.2023.115975)
Supplement: Supplementary Information (Appendix A) [file EMS184212-supplement-Supplementary_Information__Appendix_A_.pdf]

Supplementary Information for

An extended period of elevated influenza mortality risk following the main waves of influenza pandemics

Max Schroeder, Spyridon Lazarakis, Rebecca Mancy, Konstantinos Angelopoulos

Rebecca Mancy
Email: Rebecca.Mancy@glasgow.ac.uk

This PDF file includes:

Supplementary Appendices
SI References

Code and Data available at: https://zenodo.org/record/7995423

Contents

A. Data and additional data analysis 2
A.1 Sources and dataset construction 2
A.2 Influenza pandemics from 1838 to 2000 4
A.3 Influenza mortality in England and Wales relative to large municipal centres 4
A.4 Additional analysis of municipal level data for respiratory tract diseases 5
A.5 Additional analysis of the relationship between socioeconomic conditions and infant mortality 6
B. Modelling post-pandemic mortality dynamics 8
B.1 Modelling decisions 8
B.2 Details of model fitting 9
B.3 Small sample behaviour 10
B.4 Evaluation of model fit 12
B.5 The 1890-91 pandemic 14
B.6 Robustness results on variation of risk across geographies 15
B.7 Model with estimated mortality bounds 16
B.8 Different distributional assumptions 18
B.9 Additional analysis of epidemiological implications 23
C. References 24

A. Data and additional data analysis

A.1 Sources and dataset construction

City-level data were compiled from Medical Officer of Health (MOH) reports. These reports are annual administrative documents covering a range of public health-related topics at the municipal level. The first reports begin in the mid-19th century, and coverage extends to most municipalities in the UK until the early 1970s. The reports used here have been made available online on the Wellcome Collection website (Wellcome Trust, 2021). These have been partially digitised and are searchable by keyword, but quantitative data required manual transcription.

We collected influenza mortality data between 1895 and 1956 for eight large municipalities from across the UK: Belfast, Birmingham, Cardiff, Glasgow, London, Liverpool, Manchester and Sheffield. To do this, we searched relevant MOH reports for each municipality (for London, this is the London County Council). Annual statistics regarding mortality by cause of death are typically presented in tables within the reports or their appendices. Where available, we recorded numbers of deaths and population size and used these to compute mortality rates (deaths per million population). In some cases, only rates were reported, and we recorded these directly (rescaling these if necessary).

When there were no deaths from a disease in a given year, the MOH reports include a zero. However, influenza was too important a cause of death for this to apply (influenza remains a considerable cause of death into the 21st century). Nonetheless, there are a small number of missing observations in the MOH data series for influenza. These occur for two main reasons. Firstly, because the information was not available to us because the entire MOH report or the relevant parts of the report were not accessible (for example, the report was missing or only partially scanned, perhaps because parts had been damaged). In these cases, we tried to recover the information from later reports, but sometimes it was not possible to find the relevant information in any report. We believe that this type of missing information is random and so should not be a cause for concern. Secondly, there are a small number of cases in which the MOH reports combine diseases into broad categories (e.g. influenza together with pneumonia and bronchitis) so that we are unable to disentangle different causes of death effectively. There is a potential concern that this type of reporting might not be random with respect to levels of influenza mortality (e.g. in a year where there was little influenza mortality, the MOH's office might have been inclined to combine influenza with other categories). However, this is very rare and occurs at the very beginning of the sample period. Over the first decade of the 1900s, the taxonomy of disease classification becomes more standardised, and influenza as a separate cause of death becomes established across all cities by the first world war; as a result, all our main results should be unaffected. For Belfast, the 1919 MOH report is missing and later reports do not provide information on mortality in 1919. Death rates per 1,000 population for Belfast are, however, available from Beiner et al. (2009) for 1918 and 1919, with the value for 1918 being very similar to that in the MOH reports. We thus use the rate from Beiner et al. (2009) for 1919. In addition to influenza, we also compiled analogous data on other respiratory tract diseases, namely pneumonia, bronchitis, broncho-pneumonia, and other uncategorised miscellaneous respiratory diseases (usually reported in the same tables as influenza mortality). We also use infant mortality data. Infant mortality is prominently recorded in MOH Reports, and we directly transcribe the rates per 1,000 births for the years 1895-1950 (of which we use data from 1895-1917). Further details of the source of all data points are provided in the online dataset available at: https://zenodo.org/record/7995423.

Influenza mortality data for the US are taken from the Vital Statistics Rates reports compiled by the National Center for Health Statistics. Specifically, we transcribe mortality rates for the period 1900-1956 from tables in two special volumes (Grove & Hetzel, 1968; Linder & Grove, 1943) that together cover the period 1900-1960. Both volumes are available on the CDC's website at https://www.cdc.gov/nchs/products/vsus.htm. Infant mortality data for the US prior to the establishment of the Birth Registration Area (BRA) in 1915 is not available consistently (Grove, 1943). Given the rapid decline in infant mortality during the late 19th and early 20th century, using data after 1915 would likely lead to a severe underestimation of pre-pandemic infant mortality. For this reason, we exclude the US from the analysis involving infant mortality rates in Section A.5 below.

Influenza mortality data for England and Wales prior to 1901 is taken from Table 5 of Langford (2002), which covers the period 1838-1920; for most of this period, Langford compiles crude influenza mortality rates (deaths per million population) from the Registrar General Reports. Before 1900, the only exception is the period 1843-1846, for which rates for England and Wales are estimated from those documented for London, by assuming a constant ratio of influenza deaths between these two geographies, computed as the average ratio between London and England and Wales during 1840-1842. Note that Langford's values from 1911-1920 come from the Ministry of Health's *Report on the pandemic influenza* and refer to female mortality only, and we do not use these. For further details of the data sources, see Langford (2002), specifically footnote 42.

From 1901 onwards, numbers of influenza deaths by year and population data for England and Wales are taken from the 20th Century Mortality files (Office for National Statistics, 2013). These files are compiled by the Office for National Statistics using data from the Registrar General Reports. Death counts are combined with population estimates (available from the same dataset) to arrive at mortality rates. We focus on causes of death listing 'Influenza' as a primary cause (for the ICD codes included for each year, see the metadata included in the dataset at: https://zenodo.org/record/7995423). We checked agreement between the series from Langford (2002) and the 20th Century Mortality files for the overlapping period 1901-1910. Infant mortality rates for 1850-2000, of which we use the period 1895-17, are taken from the Vital Statistics tables for England and Wales (Office for National Statistics, 2022). For consistency with the infant mortality data for UK municipalities, we compute pre-pandemic infant mortality as the average rate over the period 1895-1917.

The time series of influenza death rates between 1895 and 1956 by geography are shown in Figure 1 in the main text. In Figure A-1 we plot the time series of mortality from pneumonia, bronchitis and broncho-pneumonia for the 8 UK cities between 1895 and 1950. As can be seen, there is secular downward trend in the series of these respiratory tract infections, which reflects the epidemiological transition that the UK went through during this period.

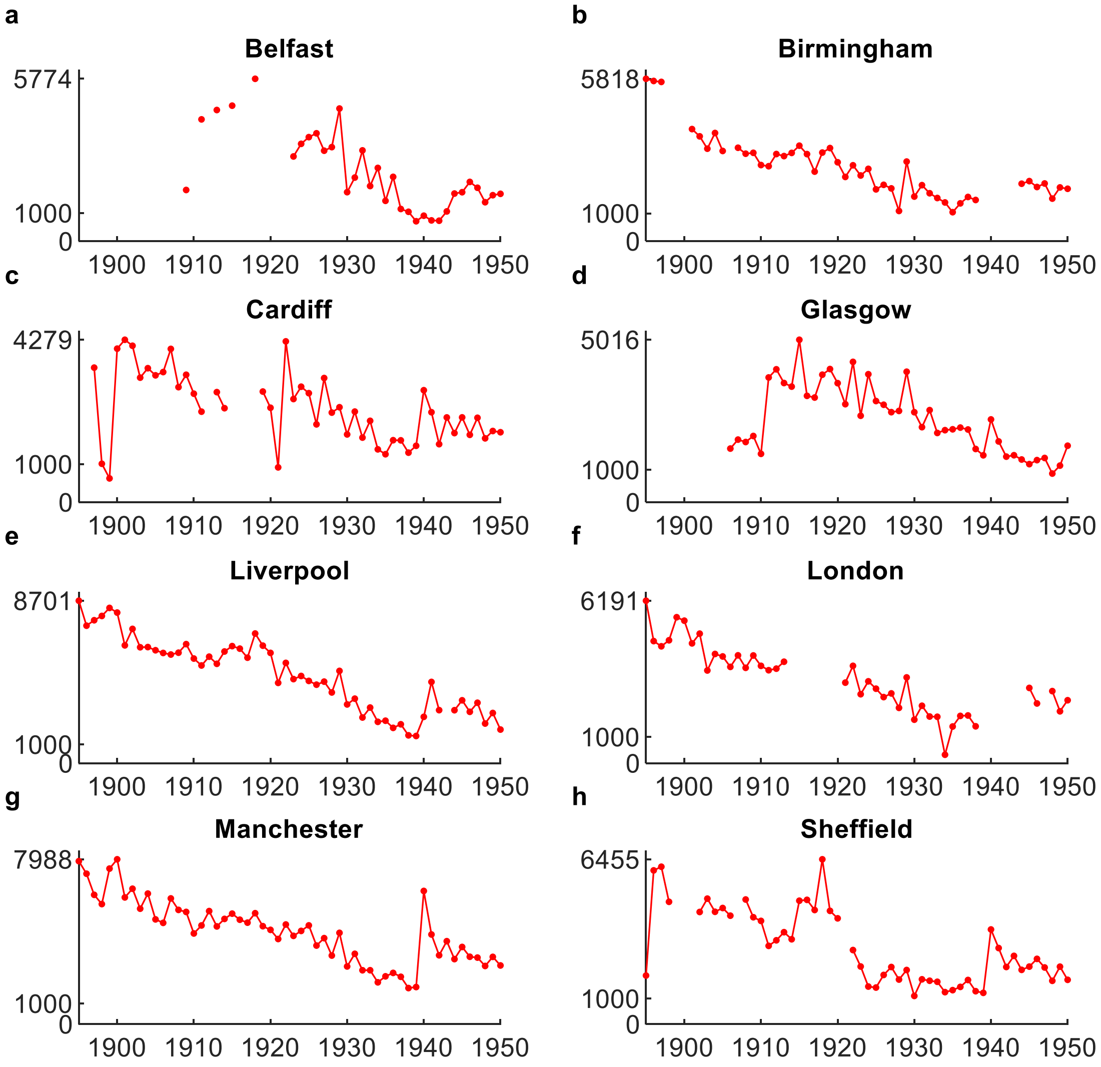

Figure A-1. Annual mortality rates from pneumonia, bronchitis and broncho-pneumonia in deaths per million population. The upper y-axis tick indicates the maximum mortality rate for each data series. Data for UK cities are taken from Medical Officer for Health reports.

A.2 Influenza pandemics from 1838 to 2000

During the period 1838-2000, there were recognised pandemics in 1847-48, 1890-91, 1918-19, 1957-59 and 1968-70 (Hill et al., 2017; Pandemic Influenza Preparedness Team, 2011). There is some ambiguity about the exact years of the pandemics, especially those during the 19th century (which may have differed between locations), and also about whether certain mortality peaks correspond to pandemics or recurrent outbreaks of earlier strains (see e.g. Hill et al., 2017 for a discussion). For example, some scholars treat 1857 as a pandemic year, but the lower mortality might instead suggest a recurrent outbreak of the 1847-48 pandemic; some experts consider the spike in mortality in 1900 as a (symptomatically mild) pandemic, but more recent sources consider it an outbreak of the 1890-91 strain. We also note that there is ongoing research on whether the 1890-91 pandemic pathogen was influenza or a coronavirus (Brüssow & Brüssow, 2021; Erkoreka et al., 2022; Ramassy et al., 2022). For further discussion of this point, see Section B.5.

A.3 Influenza mortality in England and Wales relative to large municipal centres

In Table A-1 we summarise influenza mortality for the decades surrounding the main waves of the 1918-19 pandemic for England and Wales and, on average, for the large municipal centres

in England and Wales included in our sample. We note that England and Wales generally have higher influenza mortality than the city average, but the gap closes during the period after the 1918-19 pandemic. There are many possible explanations for this effect. For example, this could reflect anomalies/spatial patterns associated with recurrent outbreaks of the 1890-91 pandemic during the earlier decades, changes in the association between influenza mortality and city living during the period, changes in the demographic make-up of populations in the studied cities relative to England and Wales and/or the effect of the different strains of influenza on different age groups (we note the unusual age distribution of mortality during the main waves of the 1918-19 pandemic, for example), or changes in the overall level of urbanisation over the period.

Table A-1: Average annual influenza mortality rate per million population during the 1918-19 pandemic and the surrounding decades for England and Wales and population weighted average across cities in England and Wales and the UK. Cities in England and Wales are: Birmingham, Cardiff, Liverpool, London, Manchester and Sheffield. Cities in the UK include all these cities as well as Belfast (Northern Ireland) and Glasgow (Scotland).

	**1898-1907**	**1908-17**	**1918-19**	**1920-29**	**1930-39**	**1940-49**
**England and Wales**	263.00	209.00	2283.00	385.00	264.00	143.00
**City average** (England and Wales)	208.99	171.27	2481.18	345.99	240.74	123.27
**City average** (UK)	199.05	166.99	2541.67	347.07	239.32	119.48
**Ratio** relative to England and Wales						
**City average** (E&W)	0.79	0.82	1.09	0.90	0.91	0.86
**City average** (UK)	0.76	0.80	1.11	0.90	0.91	0.84

A.4 Additional analysis of municipal level data for respiratory tract diseases

Analysis of mortality recorded as deaths from influenza, pneumonia, bronchitis, or broncho-pneumonia suggests that variation between cities in the mortality impact of the influenza epidemics after 1920 is similar irrespective of whether we examine mortality from influenza only or from the broader category of respiratory tract infections. For this analysis, we combine data on influenza, pneumonia, bronchitis, and broncho-pneumonia into a general category of respiratory tract diseases. Although most deaths from pneumonia, bronchitis, and broncho-pneumonia in years without influenza epidemics are probably unrelated to influenza, excess mortality in these other respiratory tract diseases during years of influenza epidemics should be largely driven by influenza. Indeed, the Medical Officers of Health note that during the influenza epidemics there was increased mortality from these other causes, arising as complications of influenza. Hence, to approximate the increased mortality during the influenza epidemic year relative to the trend, we compute relative mortality as the ratio of mortality in influenza epidemic years relative to the two surrounding years, for both influenza and the combined respiratory tract diseases. We use one year on either side of each epidemic because there can be additional epidemics if two or more years are used. In Figure A-2, we plot the relative mortality of influenza against that of the combined respiratory tract diseases, across the eight cities, for years of elevated influenza mortality during the post-1918-19 transition period. The results reveal a clear positive relationship between relative mortality in the two categories across the eight cities. Therefore, variation or similarity in the scale and persistence of outbreak risk between UK cities should not strongly depend on including or excluding in the analysis influenza-related deaths recorded as being due to other respiratory tract diseases.

We note two points of caution in relation to the interpretation of these results. First, the seasonal dynamics of respiratory tract diseases imply that we may underestimate excess mortality, both from influenza and that of the respiratory tract diseases during 'epidemic' years, because the mortality associated with the epidemic may span two calendar years. Hence, the ratio of mortality in the focal epidemic year to the previous year may be an underestimate of the excess mortality due to the epidemic. We note that although the influenza season can and sometimes

does span calendar years, the peak in the Northern Hemisphere more frequently occurs at the beginning of the calendar year (e.g. Centers for Disease Control and Prevention, 2022). Nonetheless, to the extent that seasonality also affects the respiratory tract diseases other than influenza more broadly (see e.g. Cilloniz et al., 2017 for pneumonia), if there is an underestimate of excess influenza mortality, there should also be an underestimate of excess mortality due to respiratory diseases more broadly. Therefore, although the magnitudes of excess mortality in the plots in Figure A-2 may be biased downwards, the relationship between the two excess mortalities, which is the result we draw from Figure A-2, should remain largely unaffected.

A second potential point of caution is that following a pandemic, it may be that those certifying deaths might be more likely to choose influenza as a cause of mortality relative to complications of influenza (in our case, other respiratory diseases). This is possible, although it could perhaps also be argued that they would be more mindful during or following an influenza pandemic to check which deaths are influenza-related more thoroughly, as a means to monitor and mitigate the disease. Nonetheless, if it were true that those certifying deaths were more likely to record deaths as being due to influenza close to the pandemic, and thus, relatively speaking, bias influenza mortality upwards during the early post-pandemic period, then we should see a lower slope in the relationship in Figure A-2 closer to the pandemic and a progressively steeper relationship as time goes by (a steeper relationship would suggest that for a given influenza mortality there is more respiratory disease mortality). However, the slope does not change much over time; if anything, it is shallower for the more distant epidemic of 1937. Therefore, we do not see in our results evidence of over-reporting of influenza mortality in the immediate aftermath of the pandemic relative to later years.

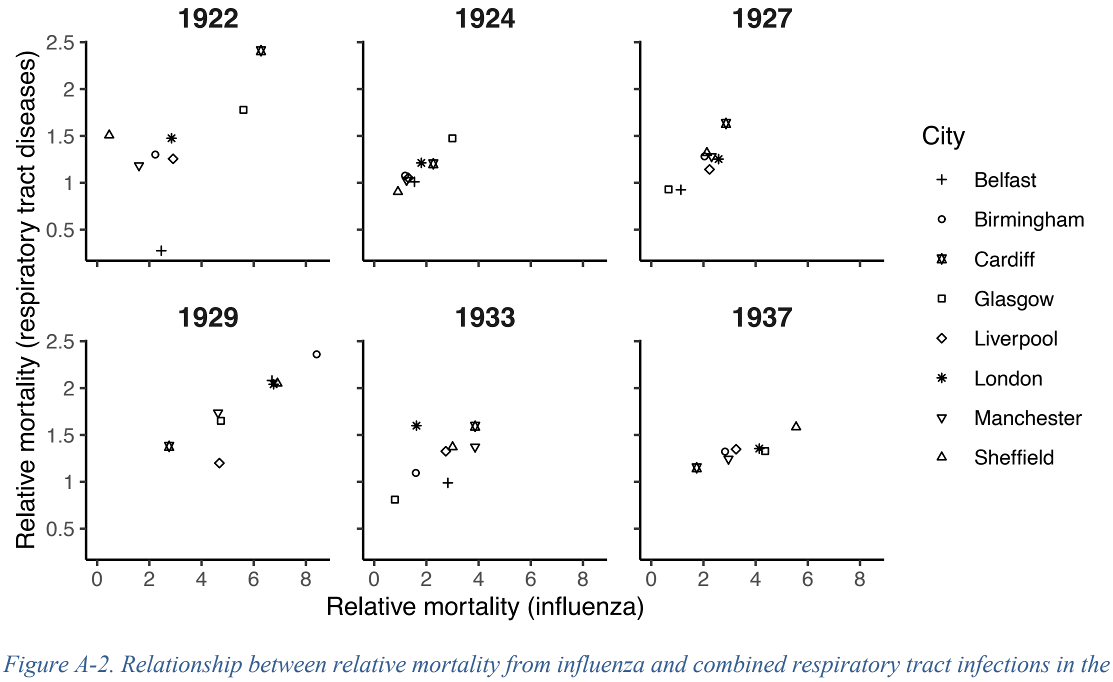

Figure A-2. Relationship between relative mortality from influenza and combined respiratory tract infections in the years 1922, 1924, 1927, 1929, 1933 & 1937. Combined respiratory tract infections include influenza, pneumonia, bronchitis and broncho-pneumonia. Relative mortality is calculated as the ratio of mortality in a given year to the average of the preceding and following year.

A.5 Additional analysis of the relationship between socioeconomic conditions and infant mortality

In the main text, we use infant mortality as a proxy for socioeconomic conditions, motivated by a large literature documenting a strong link between the two (Clay et al., 2019; Pamuk, 1988). In this section, we further validate the use of this proxy by assessing the relationship between infant mortality and measures of overcrowding in two of our municipalities.

For Glasgow, data were provided by Gillian Stewart. Overcrowding data are based on "Persons per room" which is available from primary sources during the census years of 1901, 1911 and 1921. These were reported in the "Report on Glasgow and its Sanitary Districts" from 1902, the "Report on Glasgow and its Municipal Wards" from 1912 and the "Thirteenth decennial census of the population of Scotland" from 1921. The values for intervening years are calculated using population data for each year from the MOH reports and interpolating the value for 'Windowed rooms per house' using 1901 and 1911 values from the census reports and 1921 values taken directly from the census. Infant mortality rates are available annually from the relevant MOH reports. We calculate the average for each municipal ward for both measures over the period 1903 and 1917 (note that before 1903 the information is provided by sanitary districts rather than wards). Note that between 1911 and 1912, the city expanded, and the number of wards accordingly increased, such that some averages are over a shorter period.

For London, data were provided by Siqi Qiao. Overcrowding data are based on records of the *"proportion of people living more than 2 persons to a room to the total private family population"*. These values are obtained using the *"number of people living in small tenements with more than 2 occupants per room",* which are available from the MOH reports (1901), the census report (1911) and the "*The New Survey*" (1921) (Smith, 1930), divided by census population figures. We calculate the average for each borough between 1900 and 1917. Infant mortality rates are available from the London County Council MOH reports.

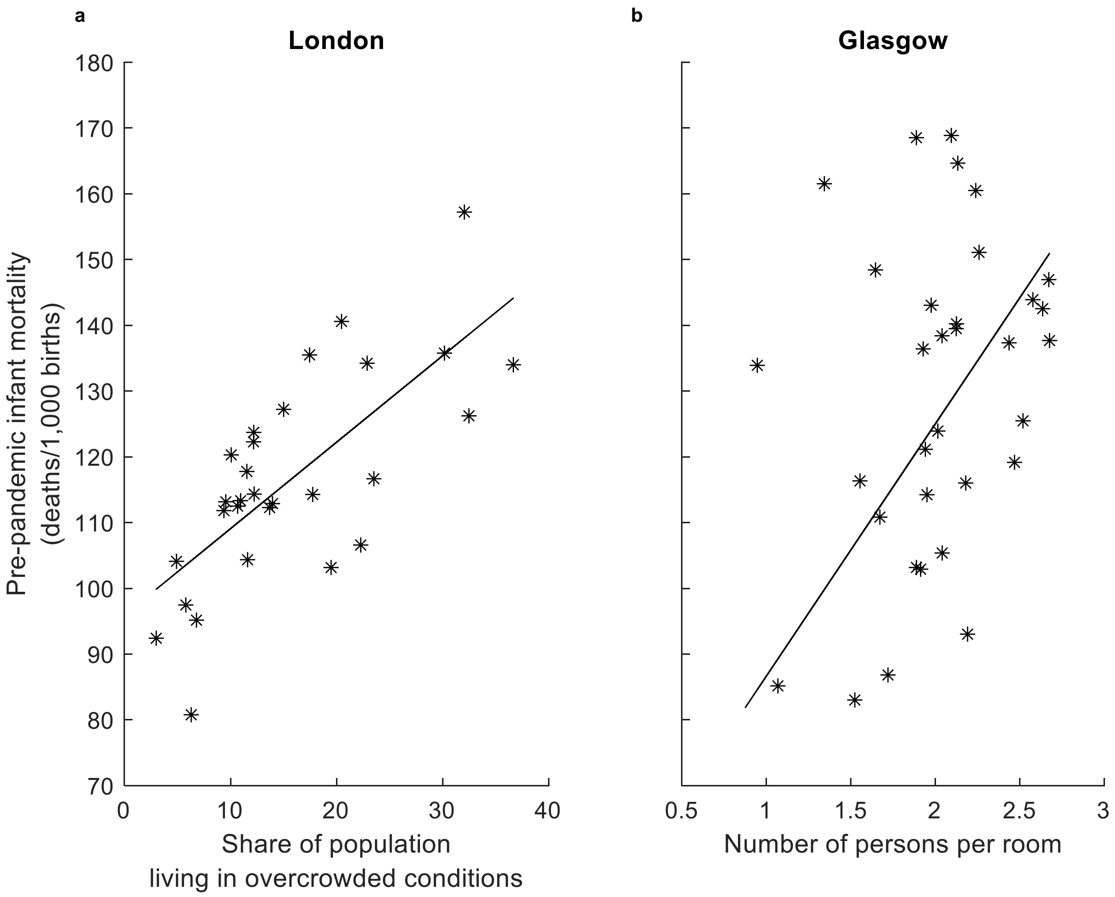

Figure A-3. Relationship between infant mortality and (a) share of population living in overcrowded conditions in the boroughs of London (1900-1917) and (b) average number of persons per room across municipal wards in the city of Glasgow (1903-1917). Line of linear best fit added for emphasis.

B. Modelling post-pandemic mortality dynamics

B.1 Modelling decisions

Statistical analysis of the dynamics of post-pandemic annual mortality rates during the transition period to background mortality requires a parsimonious model of the time evolution of over-dispersed distributions that works well in small samples.

The size of outbreaks in a given year has been shown to be highly over-dispersed and well-modelled by a fat-tailed distribution such as a bounded Pareto (Cirillo & Taleb, 2020). The bounded Pareto also offers both flexibility and tractability in the dynamic model in equations (1) – (2). The underlying distribution needs to be sufficiently flexible to capture mortality risk both during the period of relatively high mortality soon after the main waves and that of lower mortality a few decades later. The bounded Pareto distribution captures this via changes in the tail, while the mass of the distribution remains at the lower end. In addition, it has the advantage of tractability because, conditional on the bounds, changes in a single parameter capture the transition in the tail probabilities. We revisit the usefulness of the bounded Pareto distribution, relative to an alternative distributional assumption, in Section B.8.

The time evolution of mortality risk, captured in the model specification by (2), is motivated by the empirical observations shown in Figure 1. These observations imply that influenza mortality risk starts from a relatively high level, eventually converging to background mortality (i.e. a level comparable with that immediately before the relevant pandemic, as well as several decades later, e.g. associated with any low-level residual influenza A and circulating seasonal influenza B strains).

The small sample arises because each post-pandemic mortality series is bounded by the emergence of the following new pandemic. With respect to the 1847-48 pandemic, this occurred in 1890; for the 1890-91 pandemic, this occurred in 1918; and for the 1918-19 pandemic, this occurred in 1957; for the 1968-70 pandemic, our data series stops at 2000, before the next pandemic starting in 2009. For our data, this implies that our series are relatively short, consisting of up to 41 observations (note that because the post-pandemic period for the 1957-59 pandemic is less than 10 years, we do not analyse this pandemic). The small sample problem is compounded by the fact that the transition period to background mortality can be shorter than the period between pandemics, such that not all data points between two pandemics provide useful information for estimating mortality risk during the transition period. Finally, there is a small number of missing values in some time series.

The above considerations lead us to develop the parsimonious model in equations (1) – (2). We apply this model to the post-pandemic influenza mortality rates. Influenza mortality does not exhibit a secular trend over the period we study, but instead, following each pandemic, it exhibits a temporary downward trend during the endemic period, reflecting the return to background mortality (see data in Figures 1 and 2). On the contrary, deaths from the other respiratory tract diseases in Figure A-1 do demonstrate a secular downward trend, probably reflecting improvements in public health and medicine over this period as part of the epidemiological transition. To avoid problems of conflated causes and secular trends, we proceed with the statistical analysis of post-pandemic influenza mortality risk based on influenza mortality rates.

Our statistical modelling approach complements that of classic epidemiological modelling, typically in the form of susceptible-infectious-recovered (SIR) epidemiological models and their extensions (Keeling & Rohani, 2007; Mancy et al., 2017). One advantage is that it can quantify important properties of risk dynamics based on only limited information, as is often the case for historical disease episodes or wildlife diseases, while remaining agnostic about the mechanisms driving persistence. This information is useful because a range of individual and collective decisions depend less on the specific mechanisms driving persistence and more on the scale and persistence of risk, for example, in terms of assessing potential medium-run impacts on individual wellbeing, labour market and savings choices, or stress on health systems.

B.2 Details of model fitting

The model in equations (1) – (2) in the main text assumes that mortality rates after the main waves of the pandemic are outcomes drawn from a sequence of bounded Pareto distributions, where the inverse of the tail index of these distributions decays exponentially over time. The parameters $d_l > 0$ and $d_u > d_l$ scale the range of mortality rates that the model predicts. We fit the model to the data for each geographical unit conditional on its own experience of the pandemic. Hence, we choose the bounds to reflect the realised range of mortality rates for the geographical unit over the period modelled. In particular, we calibrate d_u and d_l by setting them to the maximum observed mortality rate of the main waves and to the lowest mortality experienced after the relevant pandemic and prior to the following pandemic, i.e. the lowest mortality observed in the relevant sample (see Section B.7 for estimating the bounds). Conditional on d_l and d_u, the two parameters η_0 and λ then determine the dynamics of mortality and disease outbreak risk by controlling the level and time evolution of probabilities of outcomes associated with the tail of the Pareto distributions.

We obtain λ and η_0 by maximising the likelihood function

$$L(\lambda, \eta_0) = \prod_{t=0}^{N} \frac{\left(e^{\eta_0} e^{-\lambda t}\right)^{-1} (d_l)^{\left(e^{\eta_0} e^{-\lambda t}\right)^{-1}} (\bar{d}_t)^{-\left(e^{\eta_0} e^{-\lambda t}\right)^{-1} - 1}}{1 - \left(\frac{d_l}{d_u}\right)^{\left(e^{\eta_0} e^{-\lambda t}\right)^{-1}}},$$

given a sample of mortality rates $\left(\bar{d}_t\right)_{t=0}^{N}$. To maximise the log likelihood function, we use MATLAB's fmincon routine, using a sequential quadratic programming algorithm. Derivatives are approximated by central numerical derivatives, and the relevant termination criteria are set to $1e^{-12}$. To account for potential nonconvexities and the presence of local maxima, we begin the maximisation from 1000 random seed values. Parameters of the fitted model are provided in Table B-1.

Table B-1. Model parameters for different geographies and pandemics.

	Λ	η_0	d_l	d_u
**City**				
**Belfast**	0.074	2.47	22	3644
**Birmingham**	0.132	4.137	25	2497
**Cardiff**	0.109	3.302	25	2671
**Glasgow**	0.142	4.466	24	1812
**Liverpool**	0.198	6.462	19	1775
**London**	0.094	3	23	4458
**Manchester**	0.136	4.442	23	3067
**Sheffield**	0.117	4.264	12	4550
**United States**	0.246	7.43	14	3018
**England & Wales**				
**1847-48**	0.187	5.951	2	459
**1890-91**	0.088	1.579	120	574
**1918-19**	0.102	3.308	29	3301
**1968-70**	0.127	3.922	1	148

B.3 Small sample behaviour

We examine the small sample behaviour of the maximum likelihood estimator described in Section B.2 by undertaking a Monte Carlo analysis. Assuming that the process in equations (1) – (2) is the data generating process, we generate 10,000 samples for each fitted model. We use a sample length of 37 years, which is the median transition period among the pandemics we study and also the length associated with the 1918-19 pandemic. We then fit our model to these artificial datasets. If our maximum likelihood estimation procedure is good at estimating a parameter value that is “near” the parameter of the process that generated the data, then the distribution of the estimated parameters from these 10,000 samples should be centred around the true parameters, i.e. the parameters used to simulate the data. The contour plots in Figure B-1 and Figure B-2 provide a visualisation of the joint probability density of the estimated parameters from these estimations. The joint distributions are unimodal, and in all cases, the median is effectively the same as the parameter value under the true data generating process. This informs us that if the data are generated by (1) – (2) then the parameters estimated by the maximum likelihood procedure are close to their real values.

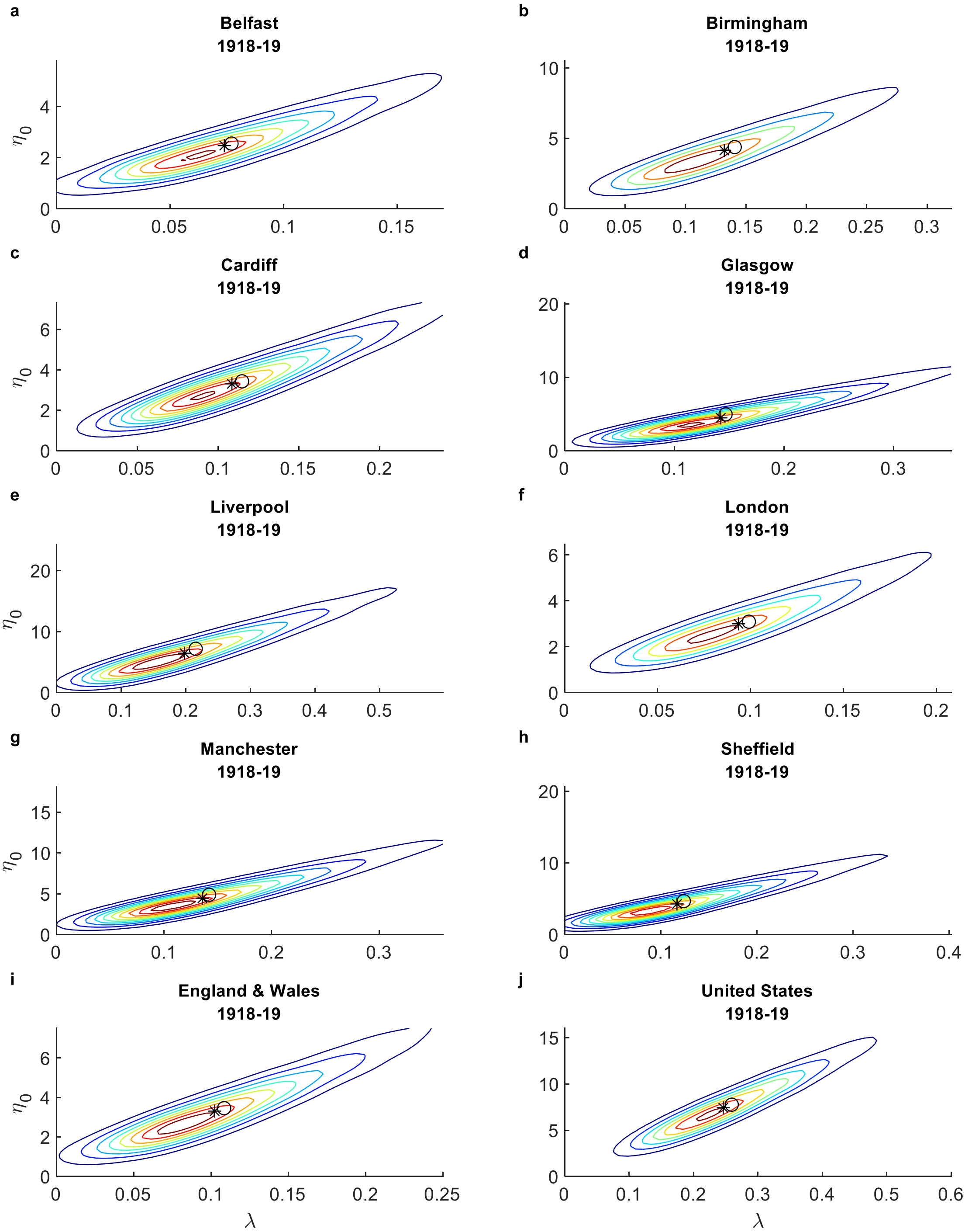

Figure B-1. Monte Carlo analysis showing the correspondence between true parameter values (stars) and medians (circles) of 10,000 Monte Carlo simulations. Contour lines represent the probability densities of combinations of $\boldsymbol{\eta_0}$ *and* $\boldsymbol{\lambda}$ *obtained from smoothed parameter kernel densities from the Monte Carlo simulations.*

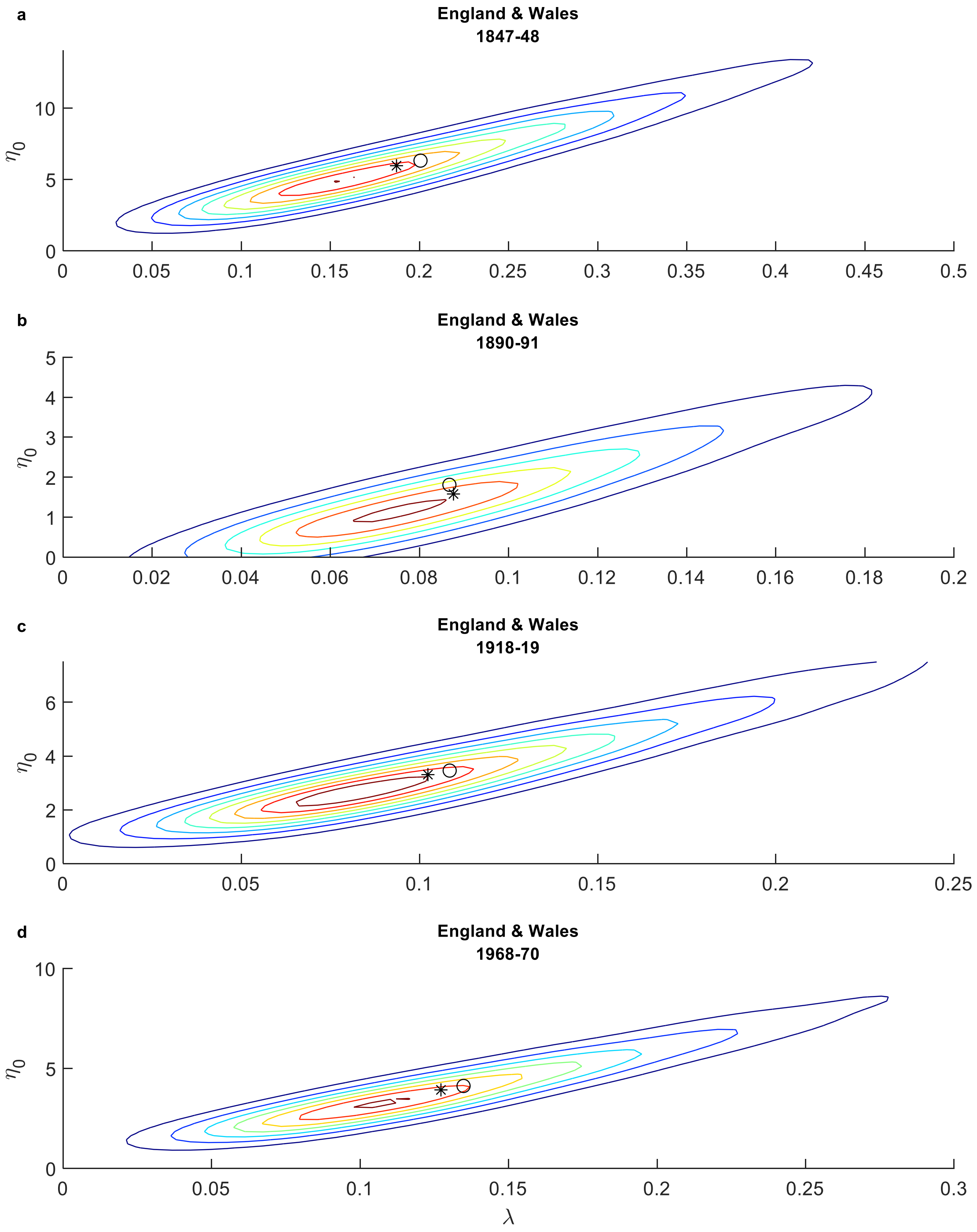

Figure B-2. Monte Carlo analysis showing the correspondence between true parameter values (stars) and medians (circles) of 10,000 Monte Carlo simulations. Contour lines represent the probability densities of combinations of $\boldsymbol{\eta_0}$ *and* $\boldsymbol{\lambda}$ *obtained from smoothed parameter kernel densities from the Monte Carlo simulations.*

B.4 Evaluation of model fit

Figure B-3 and Figure B-4 show the time series of data points overlaid on model predictions of outcomes that are based on the model fitted to each geography/pandemic. In particular, we plot the median, interquartile range and 80% interval of the estimated bounded Pareto distribution for each year. As can be seen, most observations lie within the interquartile range.

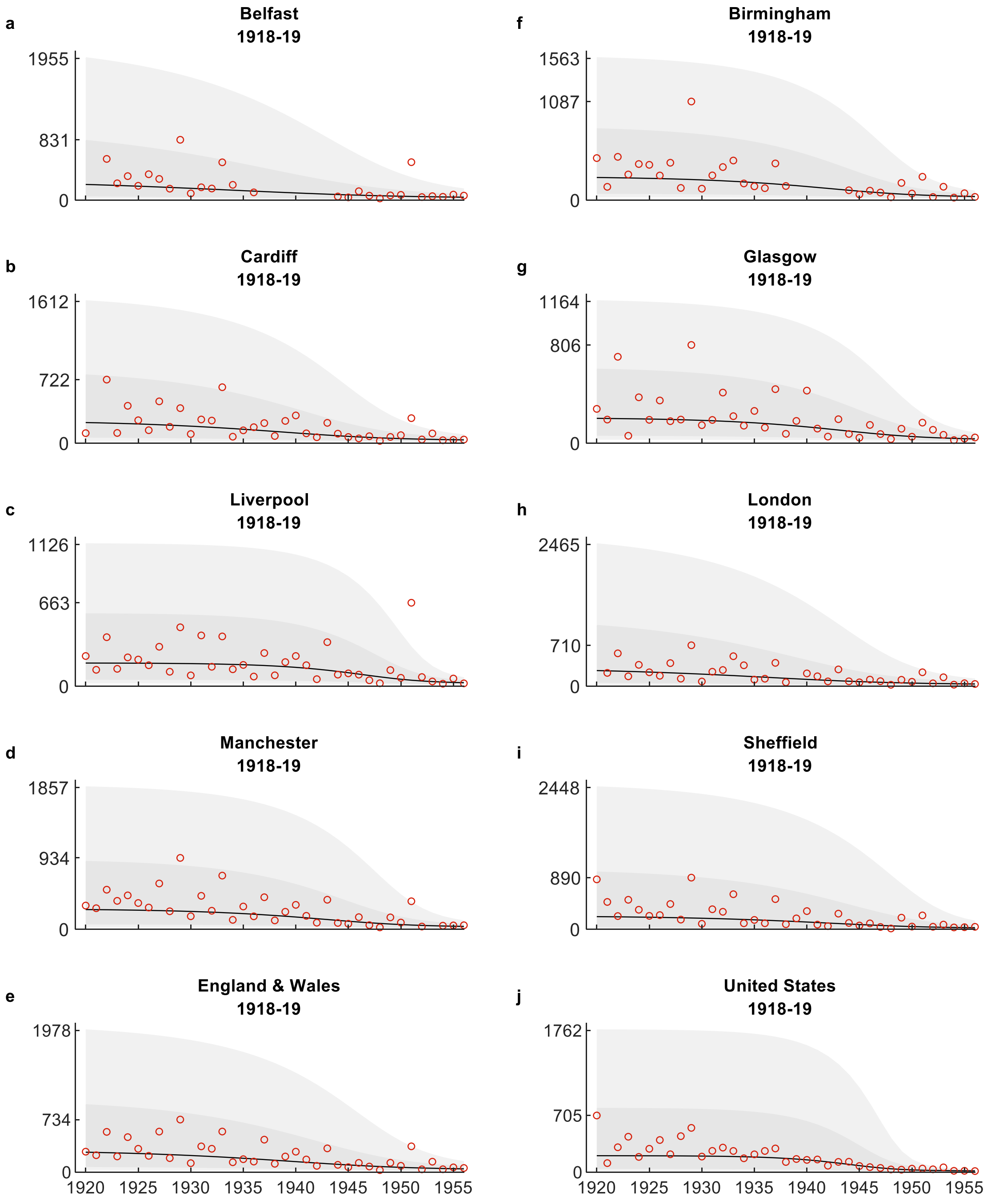

Figure B-3. Model predicted influenza mortality rates following the 1918-19 pandemic. Median (solid line), interquartile range (dark shading) and 80% prediction interval (light shading), with observed mortality rates overplotted in red. Ticks on y-axis show the upper values of the 80% prediction interval and maximum observed value in the data.

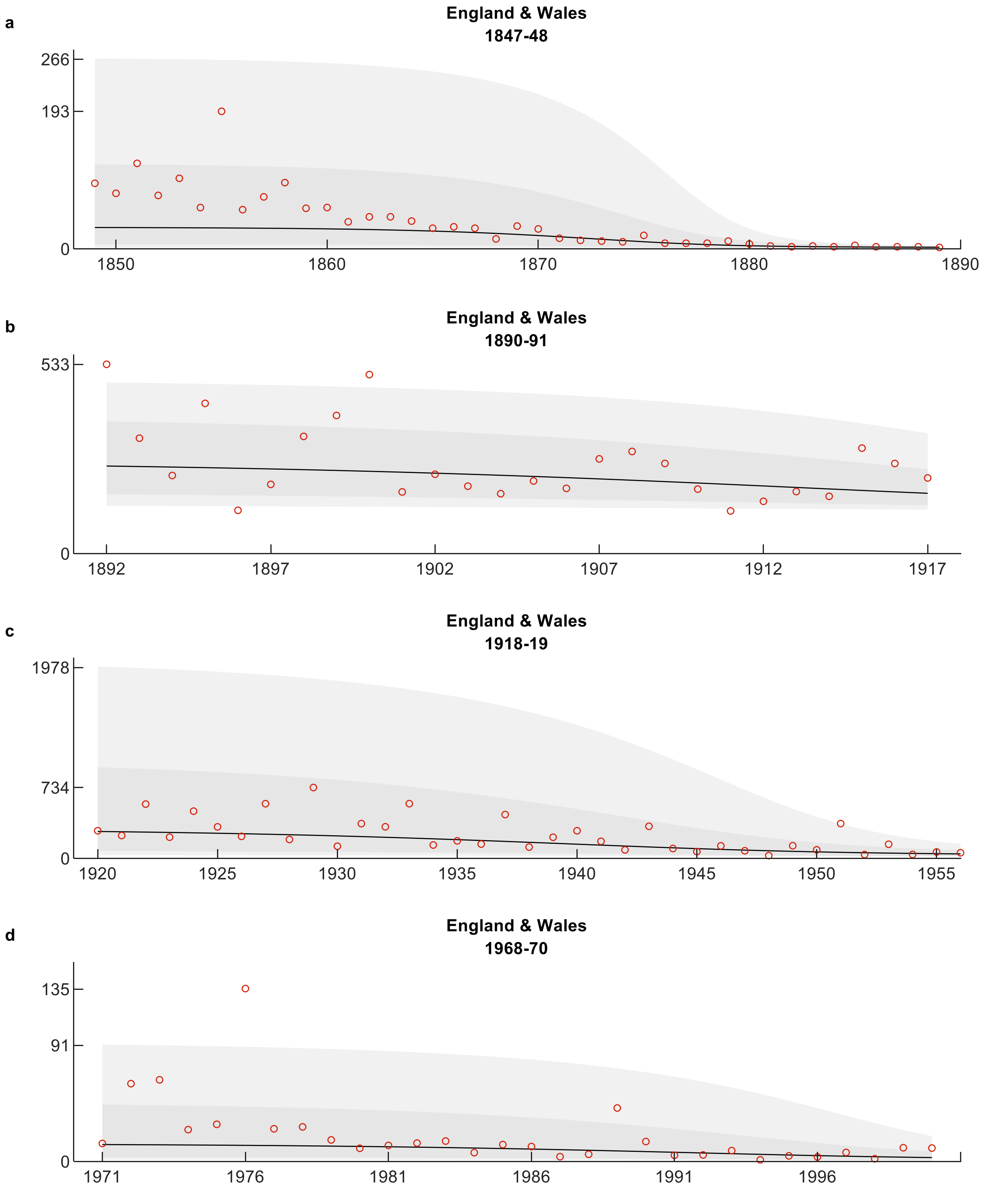

Figure B-4. Model predicted influenza mortality rates following pandemics in England & Wales. Median (solid line), interquartile range (dark shading) and 80% prediction interval (light shading), with observed mortality rates overplotted in red. Ticks on y-axis show the upper values of the 80% prediction interval and maximum observed value in the data.

B.5 The 1890-91 pandemic

The dynamic paths of risk after the 1890-91 pandemic (see Figure 4 of the main text) stand out relative to those following the other pandemics. In particular, the probability of outbreaks with high mortality relative to the main waves is substantially higher than that of the other pandemics and declines little over the two post-pandemic decades. Indeed, the high frequency of these large recurrent outbreaks is also visible in the data (Figure 2) (Brüssow, 2021 makes a similar observation). If anything, this apparent outlier strengthens our main result of persistently high

recurrent outbreak risk. However, we should note that differences in the pattern between this and the other pandemics we examine may relate to ongoing research about whether the 1890-91 pandemic was caused by influenza or a coronavirus (Brüssow, 2021; Brüssow & Brüssow, 2021; Erkoreka et al., 2022; Ramassy et al., 2022).

B.6 Robustness results on variation of risk across geographies

This section presents some robustness results with respect to the results presented in Figures 5 and 7 in the main text. Specifically, Figures B-5 and B-6 reproduce these figures, adding the 20-year slope of the decline in risk and also the case of the higher mortality threshold of 750 deaths per million. As can be seen in the figures, the results presented and discussed in the main text are robust to these changes.

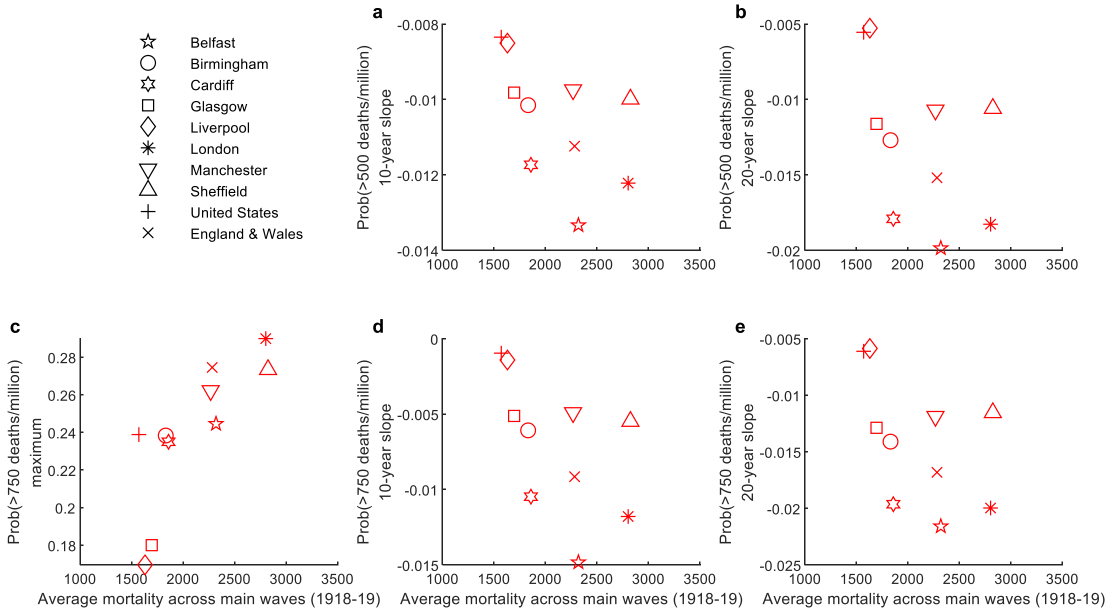

Figure B-5. Scatterplots showing the average mortality of the main waves of the 1918-19 pandemic against (a) the average slope of the outbreak risk over the first and (b) first and second post-pandemic decade for the outbreak risk mortality threshold of 500 per million; (c) the maximum outbreak risk for the mortality threshold of 750 per million; (d) the average slope of the outbreak risk over the first and (e) first and second post-pandemic decade for the outbreak risk mortality threshold of 750 per million.

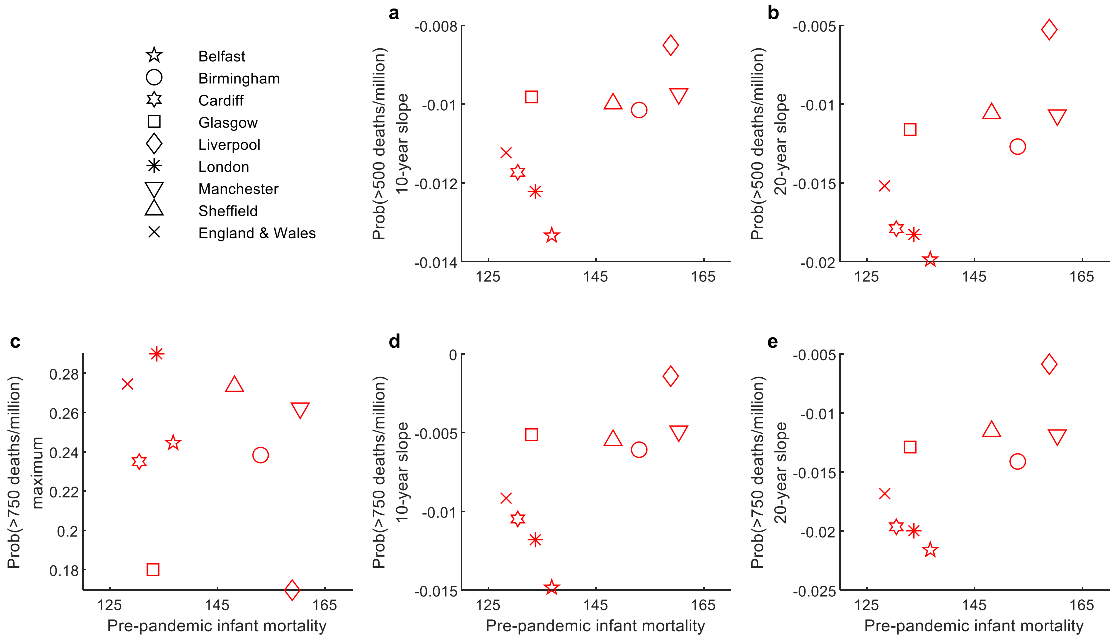

Figure B-6. Scatterplots showing pre-pandemic infant mortality against (a) the average slope of the outbreak risk over the first and (b) first and second post-pandemic decade for the outbreak risk mortality threshold of 500 per million; (c) the maximum outbreak risk for the mortality threshold of 750 per million; (d) the average slope of the outbreak risk over the first and (e) first and second post-pandemic decade for the outbreak risk mortality threshold of 750 per million.

B.7 Model with estimated mortality bounds

In our model fitting strategy in the main text, we calibrate d_l and d_u exploiting information from the main pandemic waves and background mortality to calibrate these parameters. We think that viewing the model as describing post-pandemic mortality risk conditional on the experience of the main pandemic waves is conceptually more meaningful than also estimating the mortality bounds because when analysing the post-pandemic period, the main waves have taken place, so that mortality during these is known and thus no longer a risk. Technically, however, d_l and d_u can be estimated jointly with η_0 and λ using the time series that includes the main pandemic waves. In this case, equation (3) in the main text is maximised by choosing all four parameters, following the same optimisation methods as for the model in equations (1) – (2). Figure B-7 and Figure B-8 reproduce Figure 3 and Figure 4, respectively, under this approach. As can be seen, the results are very similar, indicating that the optimiser finds that the model does achieve optimal (or near-optimal) fit when the maximum and minimum mortality are set to the maximum and minimum values observed in the data used for estimation.

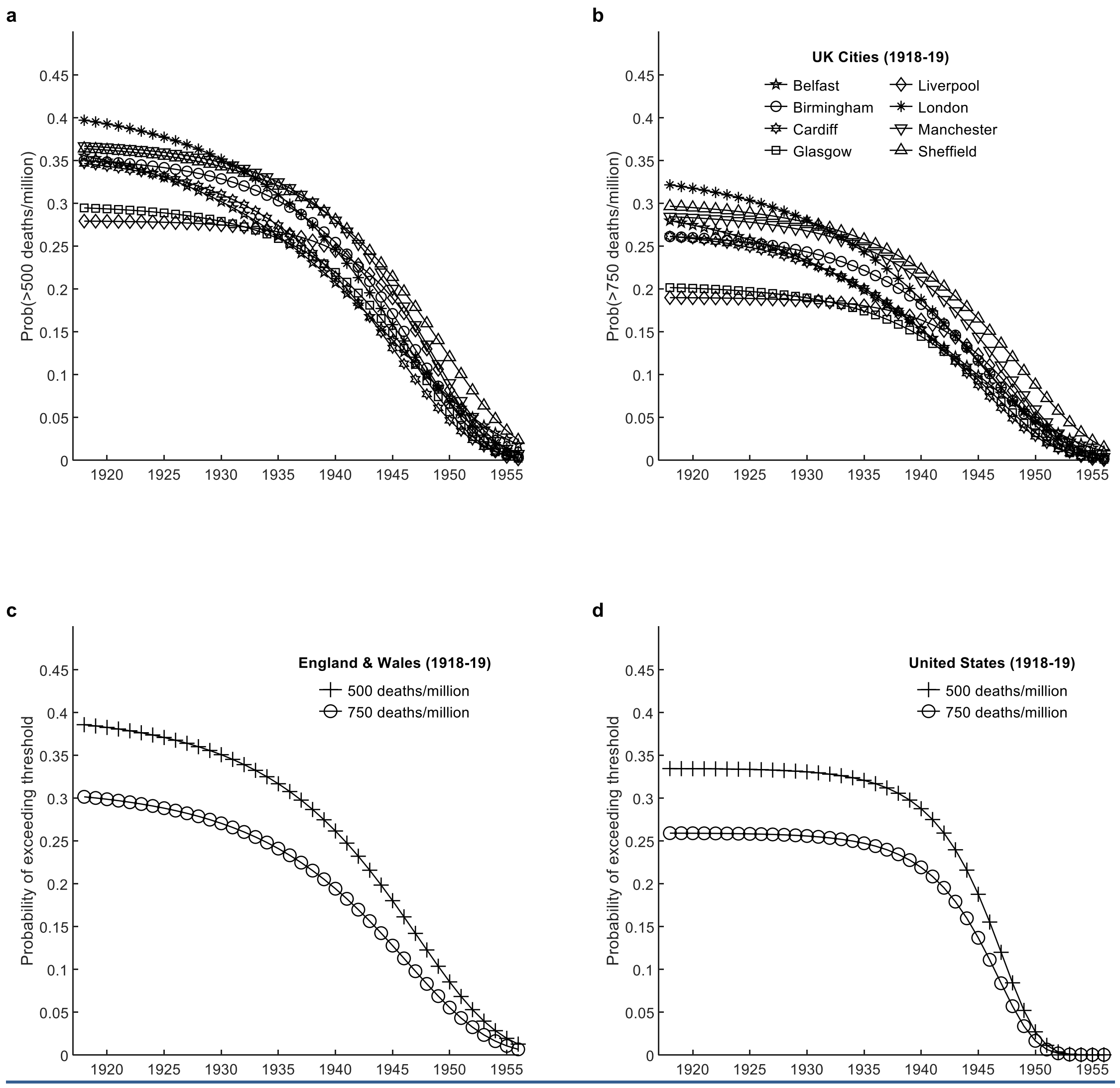

Figure B-7. Time evolution of outbreak risk following the 1918-19 pandemic from the model with estimated mortality bounds. (a)-(b) Outbreak risk computed from the model fitted to each city, and for thresholds of (a) 500 deaths per million population and (b) 1000 deaths per million population. (c)-(d) Outbreak risk computed from the model fitted to data for England and Wales and the United States, for two thresholds.

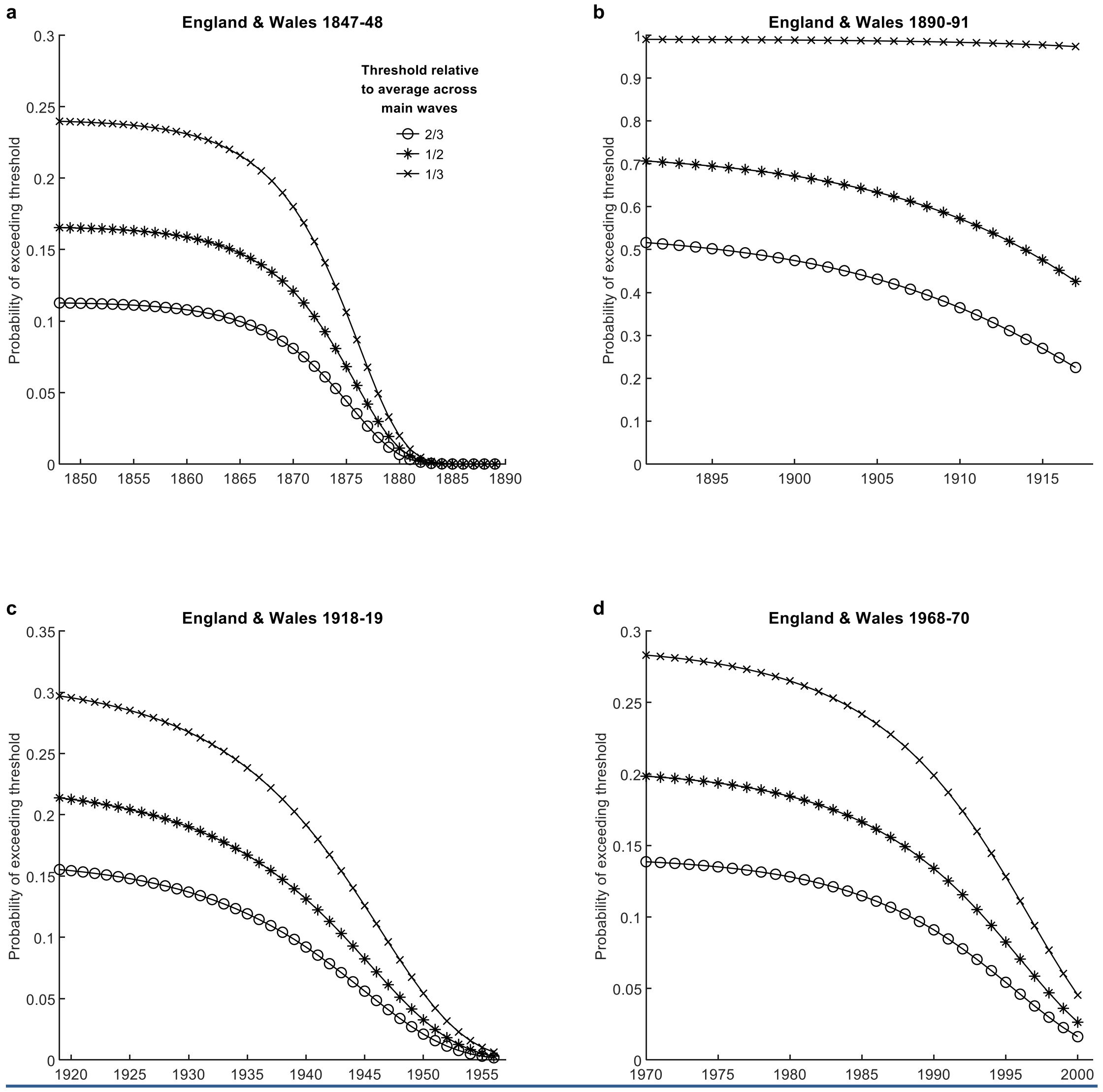

Figure B-8. Time evolution of outbreak risk following the 1847-48 and 1890-91, 1918-19 and 1968-70 pandemics in England and Wales based on the model with estimated mortality bounds, for different thresholds corresponding to proportions of the maximum mortality observed during the main waves of the corresponding pandemic.

B.8 Different distributional assumptions

To further illustrate key points of the appropriateness of the Pareto distribution, we describe and fit a model with an alternative one-parameter distribution that allows for high probabilities of outcomes associated with the tail, the one-parameter Weibull-type distribution (Alexopoulos, 2019). In this case, mortality rates are drawn from

$$d_t \sim W_t(w_t), \qquad \text{(SI 1)}$$

where $w_t \in [0,1]$, noting that the tail contracts as w_t decreases. Assume that

$$w_t = w_0 e^{-\lambda t}. \quad \text{(SI 2)}$$

Conditional on the time process in (SI 2), and thus conditional on the sequence $(w_t)_{t=0}^{N}$, d_t is independently distributed over time following (SI 1). The likelihood is given by:

$$L(\lambda, w_0) = \prod_{t=0}^{N} \frac{log^3(w_0 e^{-\lambda t})}{log(w_0 e^{-\lambda t})-2} \bar{d}_t \left(\bar{d}_t + 1\right)\left(w_0 e^{-\lambda t}\right)^{\bar{d}_t},$$

for a sample of mortality rates $(\bar{d}_t)_{t=0}^{N}$. The results from this model, shown in Figures B-9 and B-10 also reveal that mortality risk remains high for a long period after the main pandemic waves, and that its dynamic pattern is similar across geographical units. However, the predicted probabilities for disease outbreaks are higher than those in Figure 3 and Figure 4. This is an implication of the one-parameter Weibull form that delivers a fat tail by shifting the mass of the distribution away from lower numbers (Alexopoulos, 2019). Moreover, disease outbreak risk inherits a rate of rapid decline from the exponential decay of w_t, the Weibull parameter that determines the thickness of the tail. This analysis demonstrates the importance of the property of the Pareto distribution that it can accommodate a fat tail with the mass of the distribution near the lower bound of outcomes. The model fit under the one-parameter Weibull distribution assumption, seen in Figures B-11 and B-12, is not very good, with numerous outbreaks, especially after the first five years, outside of the prediction intervals.

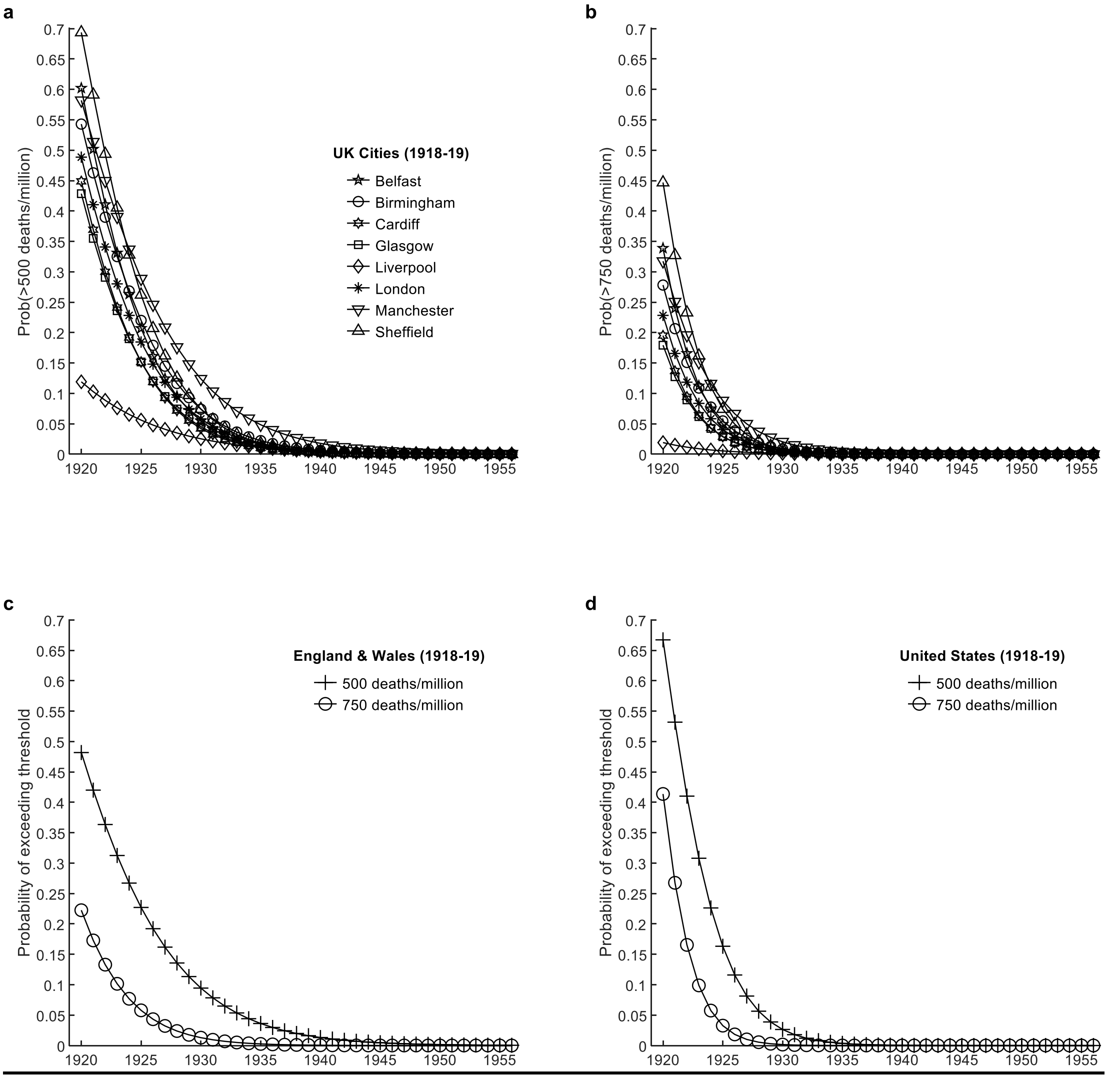

Figure B-9. Time evolution of outbreak risk following the 1918-19 pandemic produced by the one-parameter Weibull model. (a)-(b) Outbreak risk computed from the model fitted to each city, and for thresholds of (a) 500 deaths per million population and (b) 750 deaths per million population. (c)-(d) Outbreak risk computed from the model fitted to data for England and Wales and the United States, for two thresholds.

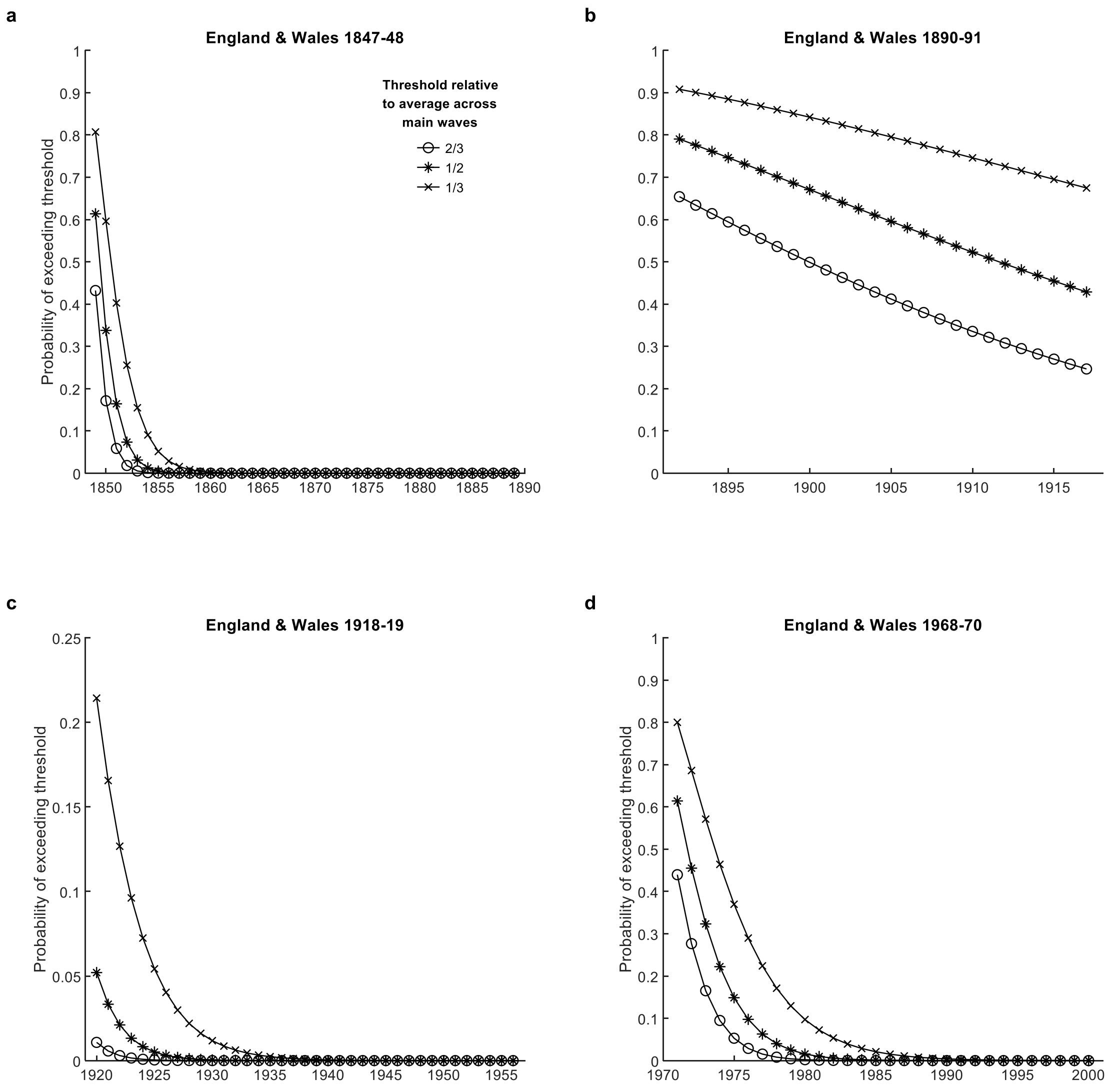

Figure B-10. Time evolution of outbreak risk following the 1847-48 and 1890-91, 1918-19 and 1968-70 pandemics in England and Wales produced by the one-parameter Weibull model, for different thresholds corresponding to proportions of the maximum mortality observed during the main waves of the corresponding pandemic.

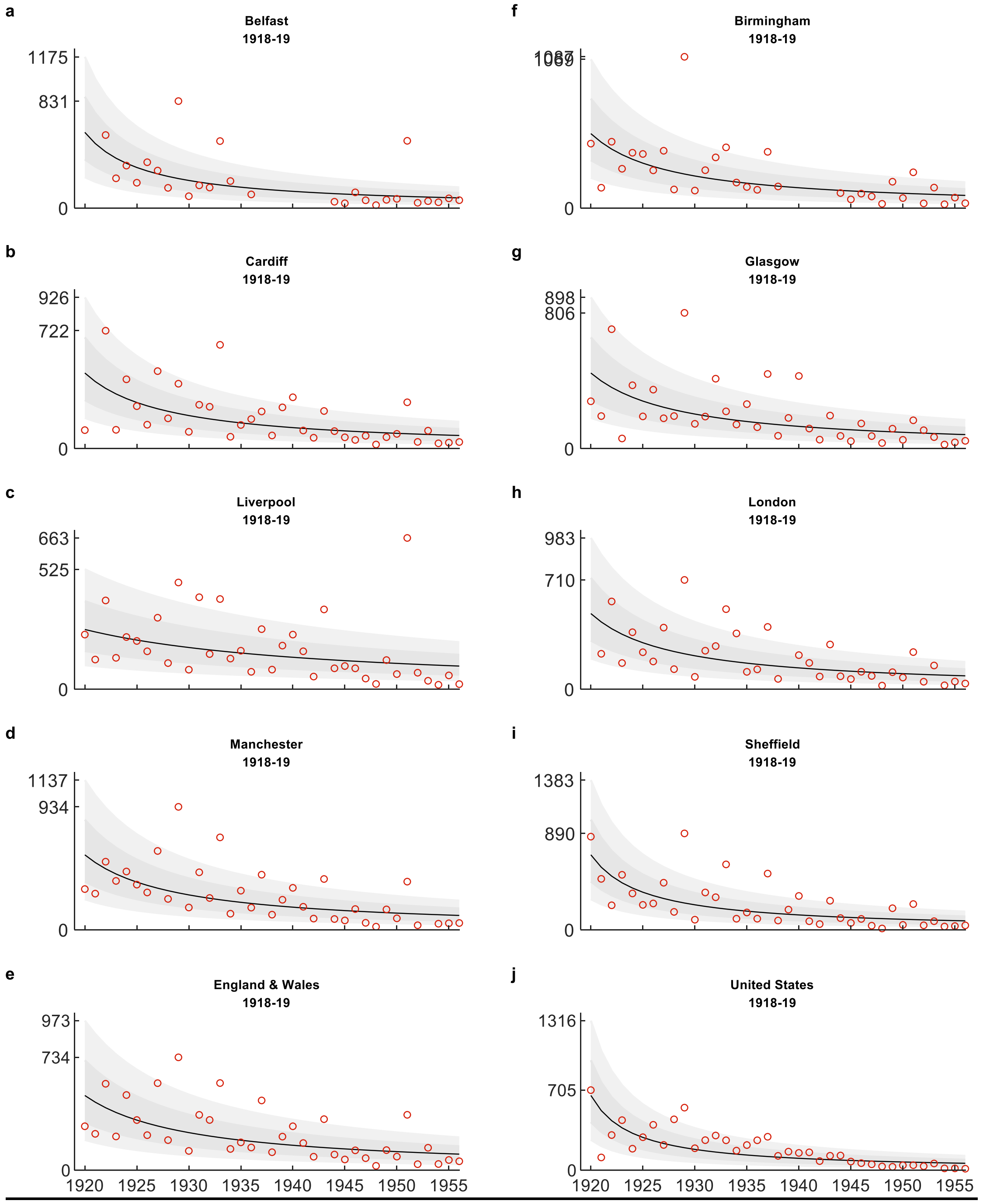

Figure B-11. Model predicted influenza mortality rates following pandemics for cities produced by the one-parameter Weibull model. Median (solid line), interquartile range (dark shading) and 80% prediction interval (light shading), with observed mortality rates overplotted in red. Ticks on y-axis show the upper values of the 80% prediction interval and maximum observed value in the data.

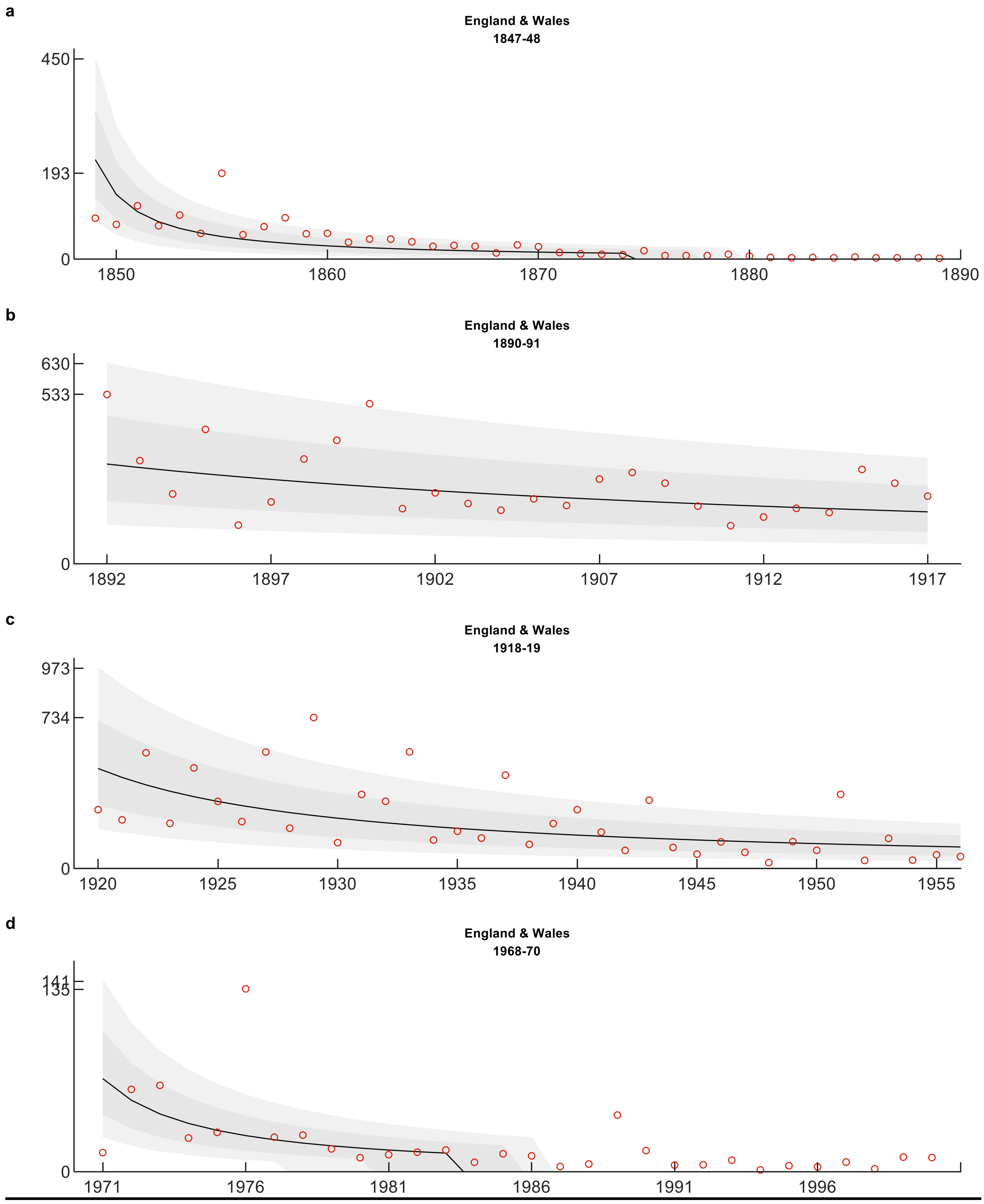

Figure B-12. Model predicted influenza mortality rates following pandemics for cities produced by the one-parameter Weibull model. Median (solid line), interquartile range (dark shading) and 80% prediction interval (light shading), with observed mortality rates overplotted in red. Ticks on y-axis show the upper values of the 80% prediction interval and maximum observed value in the data.

Although alternative distributions that allow for a concentration at lower mortality levels while also allowing for high probabilities associated with tail outcomes could be considered, these typically require more parameters to be specified (e.g. lognormal, Gaussian mixture) and require more assumptions regarding the dynamic transition. In particular, the model must specify the dynamic evolution of two or more parameters and a means to identify the specific combination of the dynamic processes of these parameters that characterises the evolution of tail probabilities and of mortality risk more generally. Data availability restricts these options. Being a one-parameter distribution conditional on the bounds for mortality rates, the bounded Pareto offers a transparent way to model the post-pandemic dynamic evolution of mortality and disease outbreak risk.

B.9 Additional analysis of epidemiological implications

Our results contribute to research on post-pandemic pathogen recurrence over the longer run. One relevant strand of research has focused on the evolution of influenza strains over the post-pandemic decades, focusing on the 1918-19 pandemic. For example, studies have identified the 1918-19 strain as the progenitor of epidemics that followed, including the 1956-57 and 1968-69 pandemics, through 'drift' (mutation) and 'shift' (antigenic acquisition or reassortment) (Morens et al., 2009; Taubenberger et al., 2019). Taking an epidemiological approach, Saglanmak et al. (2011) used data on the age distribution of excess mortality until 1937 to identify the likely timing of antigenic drift, while Morens et al. (2009) specifically identified the outbreaks in 1928-29 and 1934-36 during this period as due to drift. Morens et al. (2009) also classified as direct viral descendants of the 1918-19 strain the causative agents of 'shift' pandemics in 1957 (H2N2) and 1968 (H3N2), as well as pandemic-like events associated with intrasubtypic re-assortment in 1947 (H1N1) and 1951 (H1N1). Other work by Brüssow (2022) describes some of these epidemics, suggesting reductions in severity over time. Indeed, the time series in Figure 2 show that the more recent pandemics of 1957-59 and 1968-70 and their recurrent outbreaks resulted in lower mortality than the pandemics of the 19th and early 20th century; although this likely also arises because public health progress, it may also be partially driven by gradual reductions in the virulence of circulating strains (Morens et al., 2009). In our analysis, we treated the 1957-58 and 1968-69 pandemics separately from the 1918-19 post-pandemic period because they are typically classified as pandemics (Hill et al., 2017; Pandemic Influenza Preparedness Team, 2011). We note, however, that including them as part of the post-pandemic period of the 1918-19 pandemic strengthens our conclusion of a long period of post-pandemic recurrent outbreak risk.

Because of data limitations, we are only able to measure mortality and not morbidity over the period we study. Although elevated mortality risk clearly implies elevated morbidity risk, the scale of morbidity risk during the post-pandemic period relative to that during the main pandemic waves may show a different relationship than that observed for mortality. In fact, post-pandemic morbidity risk may even be higher relative to main wave morbidity (compared with mortality) if the pandemic strains become less virulent or treatment and prevention improve over the post-pandemic period. Although this cannot be known for all of the pandemics, the known extreme virulence of the 1918-19 strain and its apparent attenuation over time suggests that, for this pandemic, post-pandemic morbidity was likely even higher relative to main wave morbidity. In this sense, we are probably conservative in our estimates of post-pandemic health risk.

The findings of our work also raise interesting questions about how pandemic pathogens transition to endemicity. Viana et al. (2014) define an infection as endemic in a population when it is maintained without the need for external introductions. Other definitions suggest that 'an endemic infection is one in which overall rates are static – not rising, not falling' (Katzourakis, 2022) a definition that resonates with the mathematical idea of the endemic equilibrium or steady state (Kermack & McKendrick, 1932). Depending on the definition used, the point at which the pandemic pathogens studied here can be considered to be endemic differs. According to the first definition, the causal agent of the 1918-19 pandemic might, at the global scale, be considered as having been endemic more-or-less immediately following the main waves; according to the second view, the definition of endemicity would probably be fulfilled only several decades later once mortality risk was no longer in decline and incidence was indistinguishable from background influenza mortality. Other definitions could be envisaged; for example, for influenza, one might consider a novel pandemic strain to be endemic once the short-term dynamics settle into an annual pattern that resembles that of seasonal influenza (Morens & Fauci, 2007), even if overall rates remain higher. This discussion highlights the more general observation that endemic disease dynamics are typically less well understood than explosive outbreaks (Mancy et al., 2022).

C. References

Alexopoulos, A. (2019). One-Parameter Weibull-Type Distribution, Its Relative Entropy with Respect to Weibull and a Fractional Two-Parameter Exponential Distribution. *Stats*, *2*(1), 34–54. https://doi.org/10.3390/stats2010004

Beiner, G., Marsh, P., & Milne, I. (2009). Greatest killer of the twentieth century: the Great Flu of 1918-19. *History Ireland*. https://www.historyireland.com/greatest-killer-of-the-twentieth-century-the-great-flu-of-1918-19/

Brüssow, H. (2021). What we can learn from the dynamics of the 1889 'Russian flu' pandemic for the future trajectory of COVID-19. *Microbial Biotechnology*, *14*(6), 2244–2253. https://doi.org/10.1111/1751-7915.13916

Brüssow, H. (2022). The beginning and ending of a respiratory viral pandemic-lessons from the Spanish flu. *Microbial Biotechnology*, *15*(5), 1301–1317. https://doi.org/10.1111/1751-7915.14053

Brüssow, H., & Brüssow, L. (2021). Clinical evidence that the pandemic from 1889 to 1891 commonly called the Russian flu might have been an earlier coronavirus pandemic. *Microbial Biotechnology*, *14*(5), 1860–1870. https://doi.org/10.1111/1751-7915.13889

Centers for Disease Control and Prevention. (2022, September 20). *Flu season*. Influenza (Flu). https://www.cdc.gov/flu/about/season/index.html

Cilloniz, C., Ewig, S., Gabarrus, A., Ferrer, M., Puig de la Bella Casa, J., Mensa, J., & Torres, A. (2017). Seasonality of pathogens causing community-acquired pneumonia. *Respirology*, *22*(4), 778–785. https://doi.org/10.1111/resp.12978

Cirillo, P., & Taleb, N. N. (2020). Tail risk of contagious diseases. *Nature Physics*, *16*(6), 606–613. https://doi.org/10.1038/s41567-020-0921-x

Clay, K., Lewis, J., & Severnini, E. (2019). What explains cross-city variation in mortality during the 1918 influenza pandemic? Evidence from 438 U.S. cities. *Economics and Human Biology*, *35*, 42–50. https://doi.org/10.1016/J.EHB.2019.03.010

Erkoreka, A., Hernando-Pérez, J., & Ayllon, J. (2022). Coronavirus as the Possible Causative Agent of the 1889–1894 Pandemic. *Infectious Disease Reports*, *14*(3), 453–469. https://doi.org/10.3390/idr14030049

Grove, R. D. (1943). Studies In the completeness of birth registration. Part I. Completeness of birth registration in the United States, December 1, 1939 to March 31, 1940. *Vital Statistics - Special Report Series, National Office of Vital Statistics, US Public Health Service*, *17*, 223–296.

Grove, R. D., & Hetzel, A. M. (1968). *Vital statistics rates in the United States, 1940-1960* (Vol. 83). US Department of Health, Education, and Welfare, Public Health Service.

Hill, E. M., Tildesley, M. J., & House, T. (2017). Evidence for history-dependence of influenza pandemic emergence. *Scientific Reports*, *7*(1), 43623. https://doi.org/10.1038/srep43623

Katzourakis, A. (2022). COVID-19: endemic doesn't mean harmless. *Nature*, *601*(7894), 485. https://doi.org/10.1038/D41586-022-00155-X

Keeling, M. J., & Rohani, P. (2007). *Modeling Infectious Diseases in Humans and Animals* (1st ed.). Princeton University Press. http://www.amazon.ca/exec/obidos/redirect?tag=citeulike09-20&path=ASIN/0691116172

Kermack, W. O., & McKendrick, A. G. (1932). Contributions to the mathematical theory of epidemics. II. —The problem of endemicity. *Proceedings of the Royal Society of London. Series A, Containing Papers of a Mathematical and Physical Character*, *138*(834), 55–83. https://doi.org/10.1098/rspa.1932.0171

Langford, C. (2002). The age pattern of mortality in the 1918-19 influenza pandemic: an attempted explanation based on data for England and Wales. *Medical History*, *46*(1), 1–20. https://doi.org/10.1017/S002572730006871X

Linder, F. E., & Grove, R. D. (1943). *Vital statistics rates in the United States, 1900-1940*. US Government Printing Office.

Mancy, R., Brock, P. M., & Kao, R. R. (2017). An integrated framework for process-driven model construction in disease ecology and animal health. *Frontiers in Veterinary Science*, *4*(SEP). https://doi.org/10.3389/fvets.2017.00155

Mancy, R., Rajeev, M., Lugelo, A., Brunker, K., Cleaveland, S., Ferguson, E. A., Hotopp, K., Kazwala, R., Magoto, M., Rysava, K., Haydon, D. T., & Hampson, K. (2022). Rabies shows how scale of transmission can enable acute infections to persist at low prevalence. *Science*, *376*(6592), 512–516. https://doi.org/10.1126/science.abn0713

Morens, D. M., & Fauci, A. S. (2007). The 1918 influenza pandemic: Insights for the 21st century. *Journal of Infectious Diseases*, *195*(7), 1018–1028. https://doi.org/10.1086/511989

Morens, D. M., Taubenberger, J. K., & Fauci, A. S. (2009). The Persistent Legacy of the 1918 Influenza Virus. *Https://Doi.Org/10.1056/NEJMp0904819*, *361*(3), 225–229. https://doi.org/10.1056/NEJMP0904819

Office for National Statistics. (2013). *The 20th Century Mortality Files*. Https://Www.Data.Gov.Uk/Dataset/2548e46b-873e-4668-968c-25d6c155dd73/the-20th-Century-Mortality-Files.

Office for National Statistics. (2022). *Mortality in England and Wales: past and projected trends in average lifespan.* https://www.ons.gov.uk/peoplepopulationandcommunity/birthsdeathsandmarriages/lifeexpectancies/articles/mortalityinenglandandwales/pastandprojectedtrendsinaveragelifespan

Pamuk, E. R. (1988). Social-class inequality in infant mortality in England and Wales from 1921 to 1980. *European Journal of Population*, *4*(1), 1–21. https://doi.org/10.1007/BF01797104/METRICS

Pandemic Influenza Preparedness Team, U. D. of H. (2011). *Scientific Summary of Pandemic Influenza & its Mitigation: Scientific Evidence Base Review*. http://www.dh.gov.uk/en/Publicationsandstatistics/DH_125318

Ramassy, L., Oumarou Hama, H., Costedoat, C., Signoli, M., Verna, E., La Scola, B., Aboudharam, G., Barbieri, R., & Drancourt, M. (2022). Paleoserology points to Coronavirus as possible causative pathogens of the 'Russian flu'. In *Microbial Biotechnology*. John Wiley and Sons Ltd. https://doi.org/10.1111/1751-7915.14058

Saglanmak, N., Andreasen, V., Simonsen, L., Mølbak, K., Miller, M. A., & Viboud, C. (2011). Gradual changes in the age distribution of excess deaths in the years following the 1918 influenza pandemic in Copenhagen: Using epidemiological evidence to detect antigenic drift. *Vaccine*, *29*(SUPPL. 2), B42–B48. https://doi.org/10.1016/J.VACCINE.2011.02.065

Smith, H. L. (1930). *The new survey of London life & labour (Vol. 3)*. PS King & son, Limited.

Taubenberger, J. K., Kash, J. C., & Morens, D. M. (2019). The 1918 influenza pandemic: 100 years of questions answered and unanswered. In *Sci. Transl. Med* (Vol. 11). https://www.science.org

Viana, M., Mancy, R., Biek, R., Cleaveland, S., Cross, P. C., Lloyd-Smith, J. O., & Haydon, D. T. (2014). Assembling evidence for identifying reservoirs of infection. *Trends in Ecology & Evolution*, *29*(5), 270–279. https://doi.org/10.1016/j.tree.2014.03.002

Wellcome Trust. (2021, February 24). *The Medical Officer of Health reports*. https://wellcomelibrary.org/moh/about-the-reports/about-the-medical-officer-of-health-reports/
